# A time-efficient public health strategy: a systematic review and meta-regression on the comparable and dose-independent effects of sprint interval training vs. moderate-intensity continuous training for metabolic health

**DOI:** 10.3389/fpubh.2025.1708893

**Published:** 2026-01-06

**Authors:** Weibao Liang, Huaxing Zhu, Lishan Zhao, Xujie Yan, Zikun Lyu, Chuannan Liu, Wenbai Huang

**Affiliations:** 1 Guangdong Provincial Key Laboratory of Speed Capability Research, Su Bingtian Center for Speed Research and Training, School of Physical Education, Jinan University, Guangzhou, China; 2 Department of Materials Engineering, Hebei Construction Material Vocational and Technical College, Qinhuangdao, China; 3 Department of Physical Education, Hebei University of Economics and Business, Shijiazhuang, China; 4 School of Physical Education and Sports Science, Hengyang Normal University, Hengyang, China; 5 Department of Physical Education, Kunsan National University, Gunsan-si, Republic of Korea; 6 School of Physical Education and Health, Zunyi Medical University, Zunyi, China

**Keywords:** sprint interval training, moderate-intensity continuous training, high-intensity interval training, glycemic control, insulin sensitivity, metabolicsyndrome

## Abstract

**Background:**

Promoting physical activity is a public health priority for managing metabolic dysfunction, yet “lack of time” remains a major barrier to adherence. Sprint interval training (SIT) has emerged as a time-efficient alternative to traditional moderate-intensity continuous Training (MICT), but its relative effectiveness and optimal dosage are unclear. This study aimed to compare the effects of SIT vs. MICT on glycemic control and insulin sensitivity, and to investigate the influence of training dose on these outcomes.

**Methods:**

Following Preferred Reporting Items for Systematic Reviews and Meta-Analyses (PRISMA) guidelines, we conducted a systematic review and meta-analysis of randomized controlled trials (RCTs) comparing SIT (≥2 weeks) against MICT in adults with metabolic dysfunction. A random-effects model was used to determine the mean difference (MD). A series of univariable meta-regression analyses explored whether the effects were moderated by SIT dose characteristics, including intervention duration, sprint volume, and weekly training load (metabolic equivalent of task [MET]-minutes).

**Results:**

Thirteen RCTs (503 participants) were included. The pooled analysis revealed comparable effects between SIT and MICT on all primary glycemic outcomes, including glycated hemoglobin (HbA1c) (MD = −0.02%; 95% confidence interval [CI]: [−0.10, 0.07]; *p* = 0.624), homeostatic model assessment for insulin resistance (HOMA-IR) (MD = −0.08; 95% CI: [−0.27, 0.11]; *p* = 0.362), and fasting glucose (MD = 0.02; 95% CI: [−0.15, 0.20]; *p* = 0.774). Crucially, our meta-regression analyses found no statistically significant dose–response relationships. The comparable efficacy of SIT was consistent and not significantly influenced by variations in intervention duration, sprint volume, or weekly training load (*p* > 0.05 for all moderator analyses).

**Conclusion:**

Our results indicate that SIT and MICT elicit similar improvements in markers of glycemic control for adults with metabolic dysfunction. These findings suggest SIT is a viable and time-efficient alternative to MICT for individuals with limited time availability. However, variability in SIT protocols and the presence of statistical heterogeneity warrant cautious interpretation of these findings.

**Systematic review registration:**

https://www.crd.york.ac.uk/PROSPERO/view/CRD420251118188.

## 1 Introduction

The escalating global prevalence of metabolic dysfunction in the 21st century, including type 2 diabetes and prediabetes, constitutes a formidable public health challenge (1). Regular physical activity is a cornerstone of the prevention and management of these conditions, with conventional exercise guidelines advocating for at least 150 min of moderate-intensity continuous training (MICT) per week (2). Although the efficacy of MICT for improving glycemic control and insulin sensitivity is well-established, public adherence to these health recommendations remains disappointingly low. Among the barriers, a perceived “lack of time” is the most frequently cited impediment to regular exercise participation (3).

This challenge has catalyzed the exploration of time-efficient alternative exercise strategies, with high-intensity interval training (HIIT) emerging as a potent option. As a particularly intense iteration of HIIT, often distinguished from traditional HIIT (which typically involves submaximal intervals ~80–100% peak oxygen uptake [VO_2peak_]), sprint interval training (SIT) is characterized by brief, repeated ‘all-out’ or supramaximal sprints. A typical SIT session can elicit profound physiological adaptations with a substantially lower time commitment than a traditional MICT session (4, 5). Seminal studies have demonstrated that SIT can rapidly stimulate skeletal muscle mitochondrial biogenesis and improve insulin sensitivity, often yielding comparable effects to MICT despite a manifold difference in total exercise volume (6, 7).

The metabolic benefits of both SIT and MICT have garnered considerable attention, spanning improvements in cardiorespiratory fitness (8, 9), fat oxidation (10–12), blood pressure (4), glucose/insulin metabolism (13–15), post-exercise metabolism (10, 12), and hormonal regulation (16, 17). Although numerous individual randomized controlled trials (RCTs) have explored the effects of SIT in populations with metabolic dysfunction (13, 18–20), their findings have been inconsistent. Consequently, when synthesizing the available evidence, it remains unclear whether the metabolic benefits of SIT are genuinely equivalent to, or potentially greater than, those of the ‘gold standard’ MICT. Previous meta-analyses have often aggregated various forms of HIIT with SIT or have focused on narrower populations (15, 21). Therefore, a comprehensive meta-analysis is warranted not only to compare the overall effects of SIT vs. MICT, but also to investigate the key moderators and dose–response characteristics of SIT protocols that may maximize their metabolic benefits.

The present study was designed to address this knowledge gap by systematically reviewing and meta-analyzing all relevant RCTs. Its primary objectives were twofold: (1) to determine the effects of SIT on markers of insulin sensitivity and glycemic control compared to a non-exercise control; and (2) to utilize meta-regression to explore the dose–response relationship between SIT training variables and improvements in glycemic control.

## 2 Methods

The conduct and reporting of this systematic review and meta-analysis adhered strictly to the Preferred Reporting Items for Systematic Reviews and Meta-Analyses (PRISMA) 2020 statement (22). The protocol for this study was pre-registered on the International Prospective Register of Systematic Reviews (PROSPERO) under registration number: CRD420251118188.

### 2.1 Data sources and searches

A systematic search was conducted in PubMed, Embase, Web of Science, Scopus, Cochrane Library, and SPORT Discus electronic databases for articles published from database inception to June 2025. The search strategy integrated both subject headings (e.g., Medical Subject Headings [MeSH]) and free-text keywords, encompassing three core concepts: (1) sprint interval training (e.g.,” sprint interval training,”” “all-out interval training,” and “supramaximal interval training”), (2) insulin sensitivity and glycemic control (e.g., “glycemic control,” “insulin sensitivity,” “type 2 diabetes,” and “metabolic syndrome”), and (3) randomized controlled trials. The search was limited to peer-reviewed, full-text articles; gray literature, such as conference abstracts, was not included. No language restrictions were applied during the search phase. The full search strategy for PubMed is provided in the Supplementary materials.

### 2.2 Study eligibility criteria

Study inclusion was predicated on a pre-defined Population, Intervention, Comparison, Outcomes, and Study Design (PICOS) framework.

Population (P): Adults aged 18 years or older who were diagnosed with type 2 diabetes, prediabetes, or identified as being at risk for metabolic syndrome (e.g., overweight/obese individuals with metabolic abnormalities). Studies exclusively recruiting healthy, lean individuals were excluded.

Intervention (I): A longitudinal training program involving sprint interval training (SIT), defined as intermittent exercise including ‘all-out’ sprints or efforts at a supramaximal intensity (≥100% VO_2peak_).

Comparison (C): A non-exercise control group (CON) or a positive control group performing moderate-intensity continuous training (MICT).

Outcomes (O): Primary outcomes were markers of insulin sensitivity and glycemic control (e.g., homeostatic model assessment for insulin resistance (HOMA-IR), glycated hemoglobin (HbA1c), fasting glucose, fasting insulin, M-value). Secondary outcomes included other cardiometabolic markers such as lipid profiles, blood pressure, cardiorespiratory fitness (e.g., VO₂_peak_), and body composition (e.g., fat mass and body mass index [BMI]). Included studies had to report quantitative data (mean, standard deviation, and sample size) for at least one outcome of interest.

Study Design (S): Randomized controlled trials (RCTs) with an intervention period of two weeks or longer. Acute studies assessing the effects of a single exercise bout were excluded.

Based on these criteria, two independent reviewers (CL and XY) screened all study titles and abstracts. Studies assessed as relevant or unclear were subjected to full-text review. Discrepancies were resolved by a third independent reviewer (WH).

### 2.3 Data extraction

A standardized data extraction form was utilized to collect information from each included study. Two reviewers (WL and LZ) independently extracted the following data: (1) study characteristics (first author, publication year, and country); (2) participant characteristics (sample size, health status, sex, age, BMI); (3) details of the intervention protocol (modality, duration, frequency, and sprint/rest intervals); (4) details of the comparison protocol; and (5) outcome data, including sample size (N), mean, and standard deviation (SD), at pre- and post-intervention time points for all groups.

### 2.4 Risk of bias assessment

The methodological quality of each included RCT was independently assessed by two reviewers using the Cochrane Risk of Bias tool, version 2 (RoB 2) (23). This tool evaluates bias across five domains: the randomization process, deviations from intended interventions, missing outcome data, outcome measurement, and selection of the reported result. Disagreements were resolved by consensus.

Concurrently, we evaluated the certainty of evidence for each primary outcome using the Grading of Recommendations, Assessment, Development, and Evaluation (GRADE) framework. This framework rates evidence quality as high, moderate, low, or very low based on five domains (risk of bias, inconsistency, indirectness, imprecision, and publication bias).

For this review, evidence was typically downgraded one level (e.g., from “high” to” moderate”) due to the” some concerns” risk of bias judgment for most included studies (stemming from the inability to blind participants to exercise interventions) and, for some outcomes, imprecision due to wide confidence intervals.

### 2.5 Statistical analyses

Two independent, parallel meta-analyses were performed. As all outcome data were continuous and reported in similar units, the mean difference (MD) was selected as the primary effect size. A random-effects model (DerSimonian–Laird method) was employed for all analyses. We used the I^2^ statistic to quantify statistical heterogeneity between studies, which was interpreted as low (<25%), moderate (25–75%), or high (>75%). When a single study reported multiple outcomes of interest (e.g., both HbA1c and HOMA-IR), data for each outcome were extracted and included in their respective, separate meta-analyses. We did not average or prioritize outcomes from the same domain. For the head-to-head comparison of SIT vs. MICT, we conducted pre-specified subgroup analyses based on participants’ baseline health status, age, sex, intervention modality, and duration to explore potential sources of heterogeneity. Publication bias was assessed by visual inspection of funnel plots and complemented by Egger’s regression test (24). The robustness of the results was evaluated using a leave-one-out sensitivity analysis. All statistical analyses were performed using the meta-package in R version 4.5.1 software. A *p*-value < 0.05 was considered statistically significant.

To investigate the influence of SIT training characteristics on the intervention effect, we conducted several univariable random-effects meta-regression analyses. Based on the compendium of physical activities (25), we calculated the metabolic equivalent of task (MET) for each intervention protocol. We investigated four distinct SIT dose characteristics as potential moderators: (1) total intervention duration in weeks, (2) total sprint time per session in seconds, (3) total sprint time per week in seconds, and (4) cumulative weekly training load in MET-min. We examined the relationship between these moderators and the effect sizes for our primary glycemic outcomes (HbA1c, HOMA-IR, fasting insulin, and fasting glucose). A non-linear dose–response was also explored by including a quadratic term for each moderator. Bubble plots were used to visualize the results, with the size of the points weighted by the inverse of the standard error. A *p*-value < 0.05 for the regression coefficient was considered statistically significant.

## 3 Results

### 3.1 Study selection and characteristics

**Figure 1:**
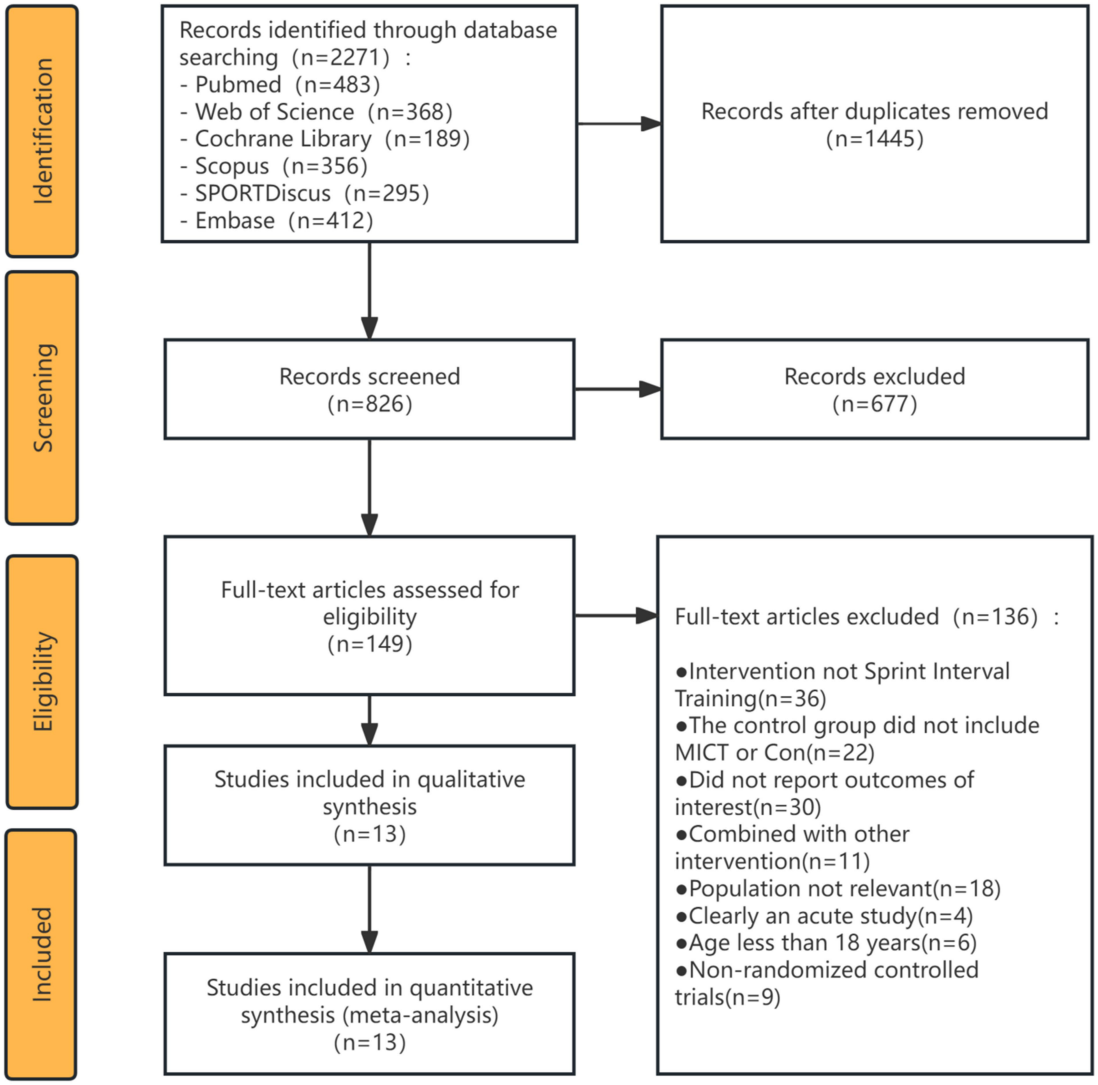
PRISMA flow diagram detailing the study selection process.

The study selection process is illustrated in the PRISMA flow diagram (Figure 1). The initial systematic database search yielded 2,271 records. After removing 1,238 duplicates, 1,033 articles remained for the screening phase. Subsequently, these articles were assessed by title and abstract, which resulted in the exclusion of 677 clearly irrelevant records. Following this, 46 full-text articles were assessed for eligibility, of which 33 were excluded for failing to meet the inclusion criteria. Ultimately, 13 randomized controlled trials (RCTs) (7, 26–37) were included in the qualitative synthesis and final meta-analysis.

### 3.2 Characteristics of the studies

#### 3.2.1 Participant characteristics

The characteristics of the 13 included studies, which encompassed 503 participants, are detailed in Table 1. These studies spanned ten different countries and were published between 2012 and 2021. The study populations primarily comprised adults diagnosed with pre-diabetes or type 2 diabetes (26–28, 30–33, 35, 36), or individuals with overweight/obesity who were at risk for metabolic syndrome (7, 29, 34, 37).

**Table 1:** Characteristics of the included studies.

Study	Country	Population	Physical Activity Level	N	Men Ratio (%)	Age (years)
Alvarez et al. (26)	Chile	Overweight or obese, pre-diabetic	Sedentary	25	0	39.4 ± 9.6
Badaam and Zingade (27)	India	Prediabetic	Physically inactive	160	100	30.9 ± 3.4
Banitalebi et al. (28)	Iran	Overweight with T2D	Sedentary	28	0	55.1 ± 5.9
Cuddy et al. (29)	USA	Low-to-moderate risk	Physically inactive	32	50	41.6 ± 10.2
Freese et al. (30)	USA	At-risk for MetS	Sedentary	45	0	51.9 ± 9.3
Gilbertson et al. (31)	USA	Pre-diabetic	Sedentary	29	Not reported	47.8 ± 4.4
Honkala et al. (32)	Finland	Normoglycemic, pre-diabetic, or T2D	Sedentary	21	61.5	48.6 ± 3.4
Motiani et al. (33)	Finland	Normoglycemic or pre-diabetic/T2D	Untrained	54	81.5	48.5 ± 1.8
Petrick et al. (34)	Canada	Overweight/obese	Insufficiently active	23	100	37.4 ± 15.1
Ruffino et al. (35)	UK	T2D	Physically inactive	16	100	55.0 ± 5.0
Sjöros et al. (36)	Finland	T2D or prediabetes	Sedentary	26	61.5	49.0 ± 4.0
Skleryk et al. (37)	Australia	Obese	Sedentary	16	100	37.8 ± 5.8
Sun et al. (7)	China/Macao	Overweight/obese	Physically inactive	28	0	21.2 ± 1.4

Data are presented as mean ± standard deviation or as specified. T2D, type 2 diabetes; MetS, metabolic syndrome.

The included studies featured a balanced sex distribution: four studies enrolled only male participants (27, 34, 35, 37), four studies enrolled only female participants (7, 26, 28, 30), four studies included mixed-sex cohorts (29, 32, 33, 36), and one study did not report the sex distribution (31). The sample size of the individual trials ranged from 16 to 160 participants. The mean age of participants varied considerably across studies, ranging from approximately 21 years (7) to 55 years (28, 35), with a focus on middle-aged adults. A common feature across all included trials was participants’ pre-intervention physical activity level, which was almost universally described as sedentary, physically inactive, or insufficiently active.

#### 3.2.2 Intervention characteristics

Details of the intervention protocols are presented in Table 2. Ten studies conducted a head-to-head comparison of SIT vs. MICT (7, 27, 29, 31–37), while the remaining three studies compared SIT against a non-exercise control (CON) group (26, 28, 30).

**Table 2:** Details of the exercise intervention protocols.

Study	Group	Exercise modality	Duration (wk)	Frequency (time/wk)	Session duration (min)	Intensity	N reps	Rep duration (s)	Work-rest ratio	Adherence (%)
Álvarez et al. (26)	SIT	Running	12	3	20	>85% HRmax	7	20–30	1:6–1:5	85
	CON	-	12	-	-	-	-	-	-	-
Badaam and Zingade (27)	SIT	Running	12	3	10 (exercise only)	All-out	4	60	1:1.05	>80
	MICT	Walking	12	5	30	Moderate	-	-	-	>80
Banitalebi et al. (28)	SIT	Cycling	10	3	~17	All-out	4	30	1:4	78
	CON	-	10	-	-	-	-	-	-	-
Cuddy et al. (29)	SIT	Cycling	8	2–4	10	All-out	2	20	1:9	89.2
	MICT	Cycling	8	3–5	25–30	40–65% HRR	-	-	-	87.8
Freese et al. (30)	SIT	Cycling	6	3	~20–35	All-out	4–8	30	1:8	-
	CON	-	6	-	-	-	-	-	-	-
Gilbertson et al. (31)	SIT	Running	16	3	~25–55	All-out	4–10	30	1:8	-
	MICT	Walking	16	3	30–60	45–55% HRR	-	-	-	-
Honkala et al. (32)	SIT	Cycling	2	3	~20–30	All-out	4–6	30	1:8	94.4
	MICT	Cycling	2	3	40–60	60% VO_2peak_	-	-	-	100
Motiani et al. (33)	SIT	Cycling	2	3	~20–30	All-out	4–6	30	1:8	-
	MICT	Cycling	2	3	40–60	60% VO_2peak_	-	-	-	-
Petrick et al. (34)	SIT	Cycling	6	3	~13–18	All-out	4–6	30	1:4	-
	MICT	Cycling	6	5	30–40	~60% Wpeak	-	-	-	-
Ruffino et al. (35)	SIT	Cycling	8	3	10	All-out	2	10–20	1:9	99
	MICT	Walking	8	5	30	40–55% HRR	-	-	-	97
Sjöros et al. (36)	SIT	Cycling	2	3	~20–30	All-out	4–6	30	1:8	-
	MICT	Cycling	2	3	40–60	60% VO_2peak_	-	-	-	-
Skleryk et al. (37)	SIT	Cycling	2	3	~15	All-out	8–12	10	1:8	-
	MICT	Cycling	2	5	30	~65% VO_2peak_	-	-	-	-
Sun et al. (7)	SIT	Cycling	12	3	20	Maintain >100 rpm	80	6	1:1.05	100
	MICT	Cycling	12	3	~63	60% VO_2peak_	-	-	-	100

SIT, sprint interval training; MICT, moderate-intensity continuous training; CON, non-exercise control; wk, week; HRmax, maximum heart rate; HRR, heart rate reserve; VO_2peak_, peak oxygen uptake; Wpeak, peak power output.

The total duration of the training programs ranged from 2 weeks (32, 33, 36, 37) to 16 weeks (31). SIT protocols typically involved 3–4 weekly sessions of “all-out” sprints, whereas MICT protocols generally consisted of 3–5 weekly sessions of 30–60 min of continuous moderate-intensity exercise (e.g., 40–65% of heart rate reserve or ~60% VO_2peak_). Regarding the SIT modality, cycling was the most common choice (10 studies), followed by running (3 studies) (26, 27, 31).

The specific SIT protocols exhibited considerable heterogeneity. The number of sprints per session ranged from as few as 2 (29, 35) to as many as 12 (37), though a protocol of 4–6 repetitions was most common. The duration of a single sprint varied from 10–30 s, with 30–s “all-out” efforts being the most frequent prescription. Work-to-rest ratios also differed substantially, from approximately 1:1.5 (27, 37) to 1:9 (29, 35).

Of the 13 included studies, seven reported adherence rates to the exercise interventions (7, 26–29, 32, 35). Overall, adherence was high for both training modalities. For SIT groups, reported adherence ranged 78–100%. For MICT groups, reported adherence was similarly excellent, ranging >80–100%. In studies reporting data for both groups, the adherence to SIT was comparable to that of MICT.

### 3.3 Risk of bias assessment

The methodological quality of each included RCT was assessed using the Cochrane RoB 2 tool (Supplementary Figures S1, S2). Overall, one study was judged to have a low risk of bias (28). Two studies were judged to be at high risk of bias due to major issues with missing outcome data (30, 31). The remaining ten studies were rated as having “some concerns” regarding risk of bias. At the domain level, the risk of bias arising from the randomization process (D1) was generally low. However, for the domain concerning deviations from intended interventions (D2), all 13 studies were rated as having “some concerns,” a common and often unavoidable issue in exercise trials where blinding is not feasible. The risk of bias due to missing outcome data (D3) was high in two studies (30, 31) but low in most others. For the domains of measurement of the outcome (D4) and selection of the reported result (D5), the risk of bias was predominantly judged as low.

### 3.4 Meta-analysis

#### 3.4.1 SIT vs. control

The meta-analysis comparing SIT with a non-exercise control group included three studies and was characterized by substantial heterogeneity (I^2^ > 80%), precluding any definitive conclusions about the absolute efficacy of SIT (Table 3). Given the small number of studies (*N* = 3) included in this specific comparison, formal subgroup or meta-regression analyses to explore the source of this high heterogeneity were not statistically feasible and were therefore not performed.

**Table 3:** Meta-analysis of the effects of SIT vs. CON.

Outcome	Number of studies	Exercise	*N*	Meta-analysis of differences in changes (SIT change-CON change)		
				Pooled difference (95%CI)	*p*	Heterogeneity l^2^, *p*
Fasting glucose (mmol/L)	3	SIT	48	−1.17 (−6.19, 3.85)	> 0.05	87.3%, 0.0004
		MICT	50			
Fasting insulin (mU/L)	3	SIT	48	−2.06 (−7.63, 3.52)	> 0.05	55.5%, 0.1060
		MICT	50			
HOMA-IR	3	SIT	48	−1.12 (−5.53, 3.28)	> 0.05	82.4%, 0.0034
		MICT	50			

The pooled difference represents the difference in the change from baseline between the SIT and CON groups. CI, confidence interval; HOMA-IR, homeostatic model assessment for insulin resistance.

#### 3.4.2 SIT vs. MICT

**Figure 2:**
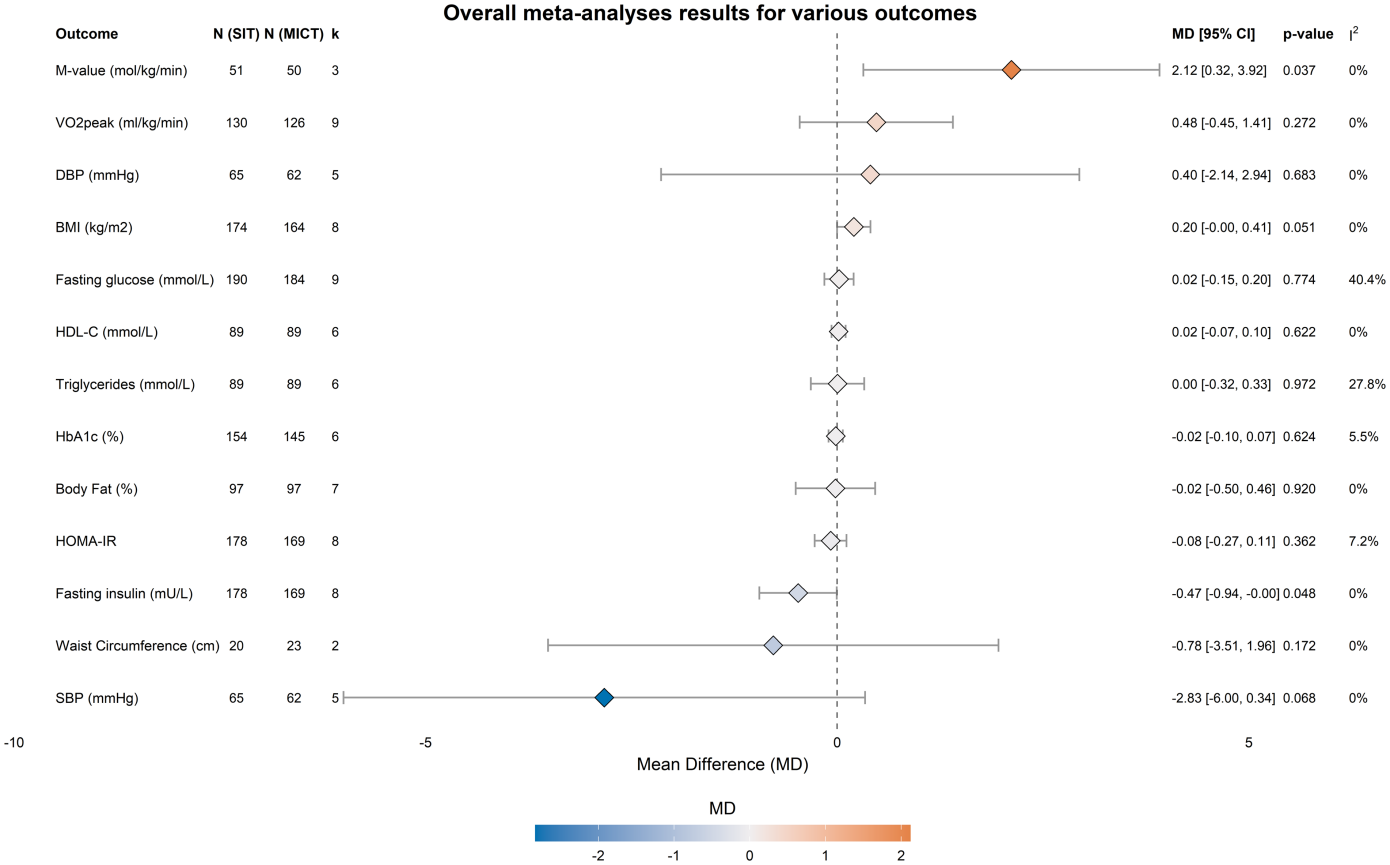
Summary forest plot of the effects of SIT vs. MICT on all outcomes. The squares and horizontal lines represent the pooled mean difference (MD) and 95% confidence intervals (CI) for each outcome. The diamond at the bottom represents the overall pooled effect, though it is not applicable in this summary plot of multiple distinct outcomes. The solid vertical line at zero represents the line of no effect.

The primary meta-analysis, comparing changes induced by SIT vs. MICT, included ten RCTs. The pooled between-group differences for all outcomes are summarized graphically in Figure 2 and detailed in Table 4.

**Table 4:** Meta-analysis of the effects of SIT vs. MICT.

Outcome	Number of studies	Exercise	*N*	Meta-analysis of pre–post changes (post value-pre value)	Meta-analysis of differences in changes (SIT change-MICT change)			GRADE
				Pooled pre–post change (95% CI)	Pooled difference (95% CI)	*p*	Heterogeneity l^2^, *p*	
Glucose metabolism								
HbA1c (%)	6	SIT	154	−0.21 (−0.34, −0.07)	−0.02 (−0.10, 0.07)	0.624	5.5%, 0.38	⨁⨁⨁
		MICT	145	−0.22 (−0.30, −0.13)				MODERATE
Fasting glucose (mmol/L)	9	SIT	190	−0.11 (−0.22, −0.01)	0.02 (−0.15, 0.20)	0.774	40.4%, 0.10	⨁⨁⨁
		MICT	184	−0.13 (−0.28, 0.02)				MODERATE
Fasting insulin (mU/L)	8	SIT	178	−0.92 (−1.73, −0.11)	−0.47 (−0.94, −0.00)	0.048	0.0%, 0.98	⨁⨁⨁
		MICT	169	−0.46 (−1.34, 0.41)				MODERATE
HOMA-IR	8	SIT	178	−0.23 (−0.50, 0.04)	−0.08 (−0.27, 0.11)	0.362	7.2%, 0.37	⨁⨁⨁
		MICT	169	−0.14 (−0.34, 0.06)				MODERATE
M-value (mol/kg/min)	3	SIT	51	5.41 (4.80, 6.02)	2.12 (0.32, 3.92)	0.037	0.0%, 0.97	⨁⨁
		MICT	50	3.28 (2.12, 4.45)				LOW
Lipid metabolism								
Triglycerides (mmol/L)	6	SIT	89	−0.11 (−0.26, 0.04)	0.00 (−0.32, 0.33)	0.972	27.8%, 0.23	⨁⨁⨁
	6	MICT	89	−0.14 (−0.32, 0.04)				MODERATE
HDL-C (mmol/L)	6	SIT	89	−0.01 (−0.09, 0.07)	0.02 (−0.07, 0.10)	0.622	0.0%, 0.55	⨁⨁⨁
	6	MICT	89	−0.02 (−0.08, 0.05)				MODERATE
Cardiorespiratory function								
VO₂_peak_ (mL/kg/min)	9	SIT	129	2.41 (0.58, 4.24)	0.48 (−0.45, 1.41)	0.272	0.0%, 0.92	⨁⨁⨁
		MICT	127	1.66 (−0.38, 3.70)				MODERATE
SBP (mm Hg)	5	SIT	65	−5.87 (−8.80, −2.94)	−2.83 (−6.00, 0.34)	0.068	0.0%, 0.94	⨁⨁⨁
		MICT	62	−3.38 (−7.27, 0.52)				MODERATE
DBP (mm Hg)	5	SIT	65	−2.13 (−4.12, −0.14)	0.40 (−2.14, 2.94)	0.683	0.0%, 0.82	⨁⨁⨁
		MICT	62	−2.65 (−5.25, −0.05)				MODERATE
Body composition								
BMI (kg/m^2^)	8	SIT	174	−0.44 (−0.78, −0.09)	0.20 (−0.00, 0.41)	0.051	0.0%, 1.00	⨁⨁⨁
		MICT	164	−0.60 (−1.06, −0.14)				MODERATE
Waist Circumference (cm)	2	SIT	20	−1.22 (−1.77, −0.68)	−0.78 (−3.51, 1.96)	0.172	0.0%, 0.95	⨁
		MICT	23	−0.45 (−3.72, 2.83)				Very Low
Body fat (%)	7	SIT	97	−0.58 (−1.07, −0.08)	−0.02 (−0.50, 0.46)	0.920	0.0%, 1.00	⨁⨁⨁
		MICT	97	−0.58 (−0.95, −0.20)				MODERATE

Data are presented as pooled mean differences (MDs) with 95% confidence intervals (CIs). The ‘Meta-analysis of pre–post changes’ columns show the pooled within-group change from baseline for SIT and MICT separately. The ‘Meta-analysis of differences in changes’ column represents the primary comparison, indicating the net difference in effect between SIT and MICT. N, number of participants; I^2^, I-squared statistic for heterogeneity; HbA1c, glycated hemoglobin; HOMA-IR, homeostatic model assessment for insulin resistance; HDL-C, high-density lipoprotein cholesterol; VO_2peak_, peak oxygen uptake; SBP, systolic blood pressure; DBP, diastolic blood pressure; BMI, body mass index.

Overall, the two training modalities elicited comparable effects across a wide range of cardiometabolic outcomes. No significant between-group differences were observed for the primary markers of glycemic control. Specifically, the pooled effect for glycated hemoglobin (HbA1c) was non-significant (MD = −0.02%; 95% CI [−0.10, 0.07]; *p* = 0.624), as were the effects for fasting glucose (MD = 0.02 mmol/L; 95% CI [−0.15, 0.20]; *p* = 0.774) and HOMA-IR (MD = −0.08; 95% CI [−0.27, 0.11]; *p* = 0.362). Notably, statistical heterogeneity for these primary outcomes was low to moderate (I^2^ = 5.5% for HbA1c, 40.4% for fasting glucose, and 7.2% for HOMA-IR, respectively). Furthermore, no statistically significant differences were found between SIT and MICT for any secondary outcomes, including measures of lipid metabolism, cardiorespiratory function, or body composition (*p* > 0.05 for all).

While the pooled analysis initially suggested a statistically greater reduction in fasting insulin following SIT (MD = −0.47 mU/L; 95% CI [−0.94, −0.00]; *p* = 0.048), this finding was of marginal significance, with the confidence interval just touching zero, suggesting this result should be interpreted with caution. Similarly, the observed improvement in the M-value (MD = 2.12 mg/kg/min; 95% CI [0.32, 3.92]; *p* = 0.037) was not robust, losing statistical significance during the leave-one-out sensitivity analysis.

The certainty of evidence for most primary outcomes was rated as moderate using the GRADE framework. Collectively, these results indicate that SIT and MICT produce broadly similar metabolic improvements. This highlights the importance of exploring the heterogeneous training protocols more deeply, as the overall pooled analysis may obscure critical dose–response relationships that determine the specific conditions under which one modality might be superior.

### 3.5 Dose–response meta-regression analysis

We performed four separate univariable meta-regression analyses to determine different aspects of the training dose influenced the comparative effect of SIT.

**Figure 3:**
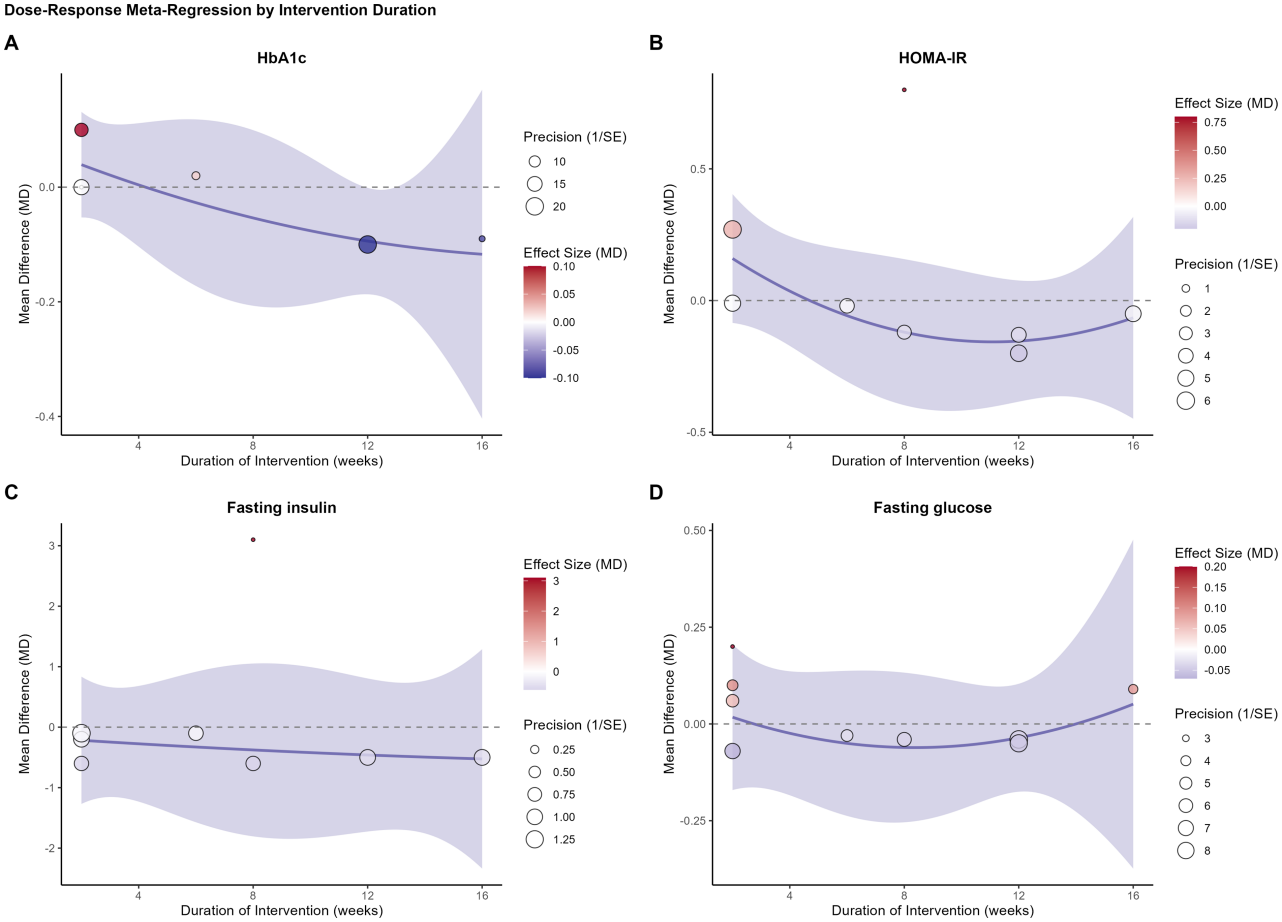
Bubble plots illustrating the univariable meta-regression analysis of the association between intervention duration (weeks) and the mean difference (MD) in glycemic outcomes between SIT and MICT. **(A)** Glycated hemoglobin (HbA1c); **(B)** HOMA-IR; **(C)** Fasting insulin; **(D)** Fasting glucose. Each bubble represents an individual study, with the size of the bubble proportional to the study’s weight (inverse of the standard error). The solid line represents the estimated regression line, and the shaded area represents the 95% confidence interval.

**Figure 4:**
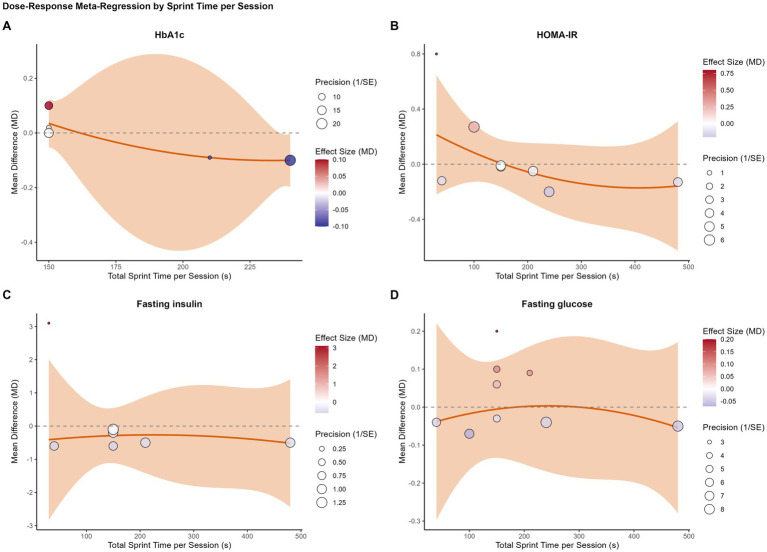
Bubble plots illustrating the univariable meta-regression analysis of the association between total sprint time per session (seconds) and the mean difference (MD) in glycemic outcomes between SIT and MICT. **(A)** Glycated hemoglobin (HbA1c); **(B)** HOMA-IR; **(C)** Fasting insulin; **(D)** Fasting glucose. Each bubble represents an individual study, with the size of the bubble proportional to the study’s weight (inverse of the standard error). The solid line represents the estimated regression line, and the shaded area represents the 95% confidence interval.

**Figure 5:**
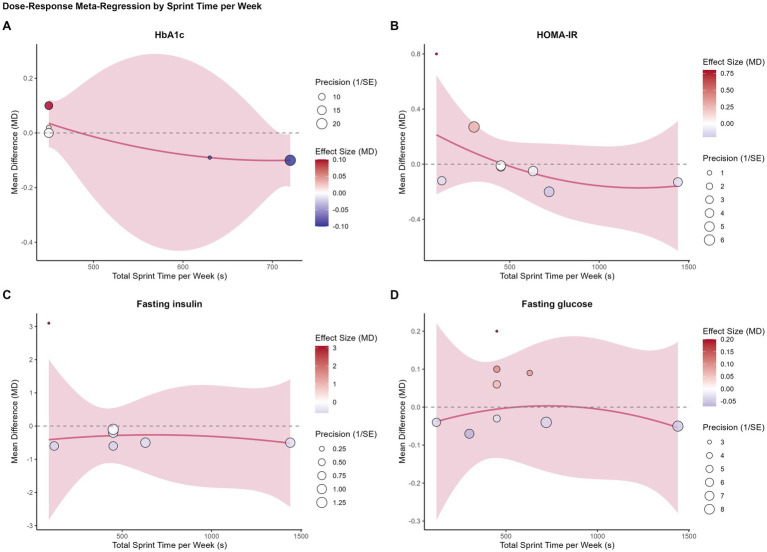
Bubble plots illustrating the univariable meta-regression analysis of the association between total sprint time per week (seconds) and the mean difference (MD) in glycemic outcomes between SIT and MICT. **(A)** Glycated hemoglobin (HbA1c); **(B)** HOMA-IR; **(C)** Fasting insulin; **(D)** Fasting glucose. Each bubble represents an individual study, with the size of the bubble proportional to the study’s weight (inverse of the standard error). The solid line represents the estimated regression line, and the shaded area represents the 95% confidence interval.

**Figure 6:**
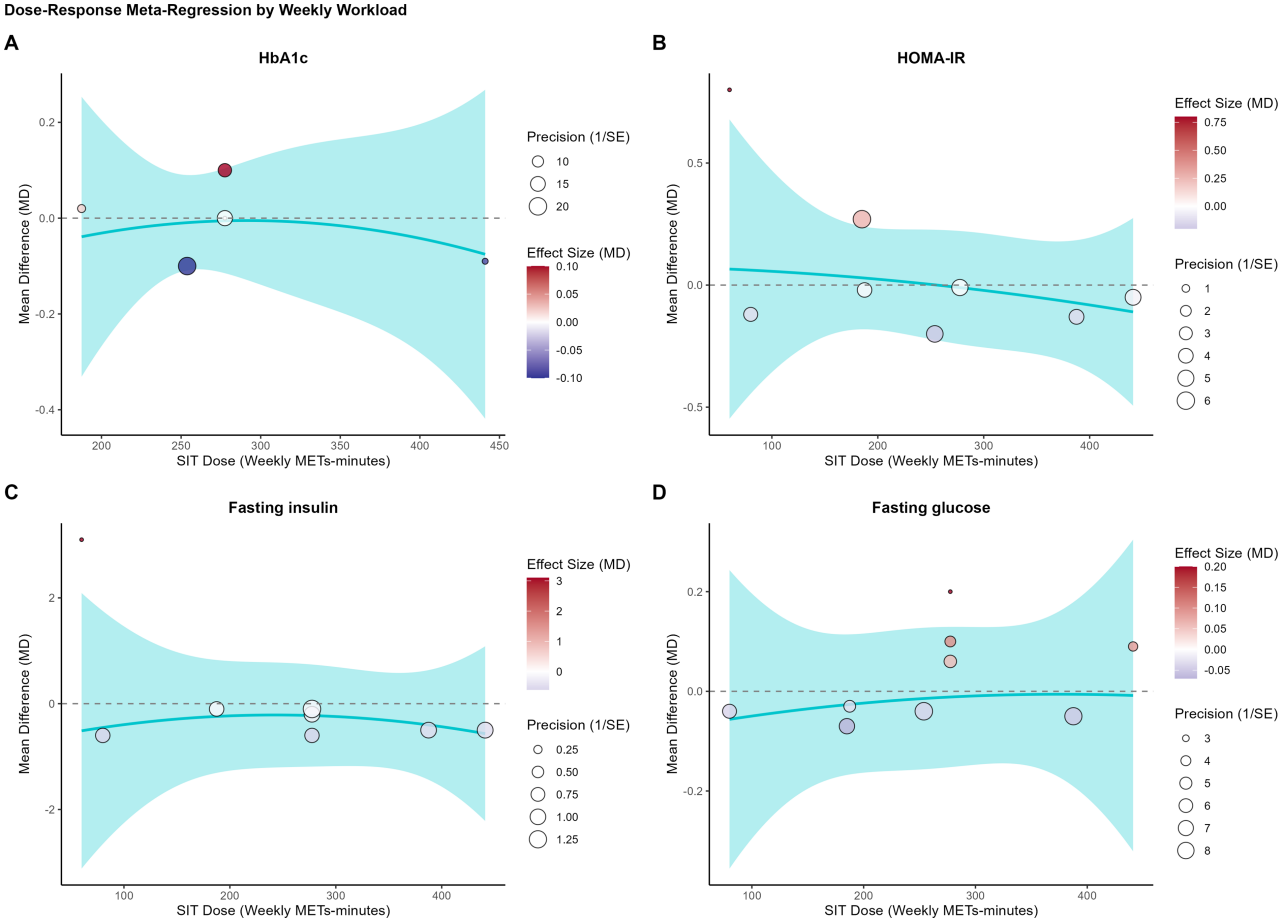
Bubble plots illustrating the univariable meta-regression analysis of the association between cumulative weekly training load (MET-minutes) and the mean difference (MD) in glycemic outcomes between SIT and MICT. **(A)** Glycated hemoglobin (HbA1c); **(B)** HOMA-IR; **(C)** Fasting insulin; **(D)** Fasting glucose. Each bubble represents an individual study, with the size of the bubble proportional to the study’s weight (inverse of the standard error). The solid line represents the estimated regression line, and the shaded area represents the 95% confidence interval.

The results were consistent across all models. No statistically significant dose–response relationships were found for any of the primary glycemic outcomes. Specifically, neither intervention duration (weeks) (Figure 3), total sprint time per session (Figure 4), total sprint time per week (Figure 5), nor the cumulative weekly training load (MET-minutes) (Figure 6) was a significant predictor of the mean difference in HbA1c, HOMA-IR, fasting insulin, or fasting glucose. The detailed regression coefficients, 95% CIs, k, and specific *p*-values for all linear models are presented in Supplementary Table S4 (all *p*-values > 0.20).

These results suggest that, within the dose range of the analyzed studies, the comparable efficacy of SIT and MICT is consistent and not dependent on the specific training volume or duration.

### 3.6 Subgroup and sensitivity analysis

**Figure 7:**
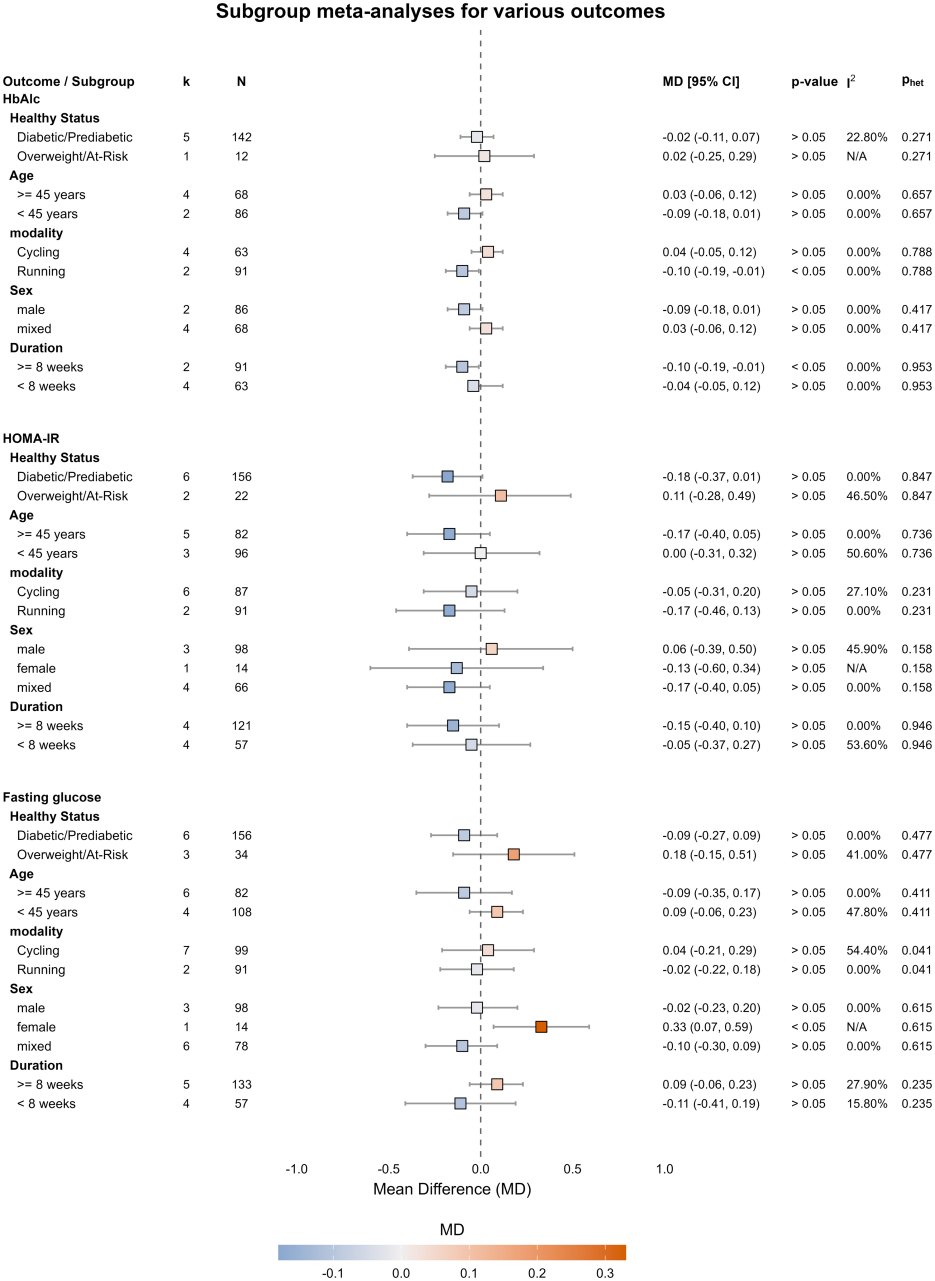
Forest plot of subgroup analyses for the effect of SIT vs. MICT. Subgroup analyses are shown for the primary outcomes of HbA1c, HOMA-IR, and fasting glucose, based on baseline health status, age, exercise modality, sex, and intervention duration. Data are presented as Mean Difference (MD) with 95% Confidence Intervals (CI). The *p*-value for the moderation effect tests for significant differences between subgroups.

To explore potential sources of heterogeneity and moderation effects, we conducted subgroup analyses based on several pre-specified factors: baseline health status, age, exercise modality, sex, and intervention duration. The detailed results are presented in Supplementary Table S2. The results of these analyses for the effect of SIT vs. comparator interventions on HbA1c and HOMA-IR are presented in Figure 7. For HbA1c, both the exercise modality (p for moderation effect = 0.040) and the intervention duration (p for moderation effect = 0.040) were found to be significant effect modifiers. Specifically, running-based SIT (MD = −0.10, 95% CI [−0.19, −0.01]) and interventions lasting eight weeks or longer (MD = −0.10, 95% CI [−0.19, −0.01]) showed a statistically significant benefit in reducing HbA1c compared to control interventions, whereas cycling-based and shorter-duration interventions did not.

A leave-one-out sensitivity analysis confirmed the robustness of most findings. However, the significant effect on the M-value was no longer statistically significant after the removal of one study (26), suggesting this specific finding should be interpreted with caution. In contrast, the significant finding for fasting insulin remained robust. The full set of sensitivity analysis plots is provided in Supplementary Figures S3–S18.

### 3.7 Publication bias

Visual inspection of the funnel plots for the primary outcomes, provided in Supplementary Figures S19–S31, did not suggest significant asymmetry. This was supported by Egger’s regression tests, which were non-significant for all main outcomes (*p* > 0.05), indicating a low risk of publication bias influencing the results of this meta-analysis.

## 4 Discussion

### 4.1 Summary of principal findings

This systematic review and meta-analysis conducted a comprehensive evaluation of SIT compared to MICT for adults with metabolic dysfunction. Our principal finding is that, in a head-to-head comparison with the currently recognized “gold standard” exercise modality, SIT demonstrates strong value as a time-efficient and equally effective alternative. The two training modalities elicited comparable effects on a wide range of indicators, including HbA1c, HOMA-IR, fasting glucose, cardiorespiratory fitness, and body composition. While an initial pooled analysis suggested a marginal advantage of SIT in reducing fasting insulin, this finding was not robust.

**Figure 8:**
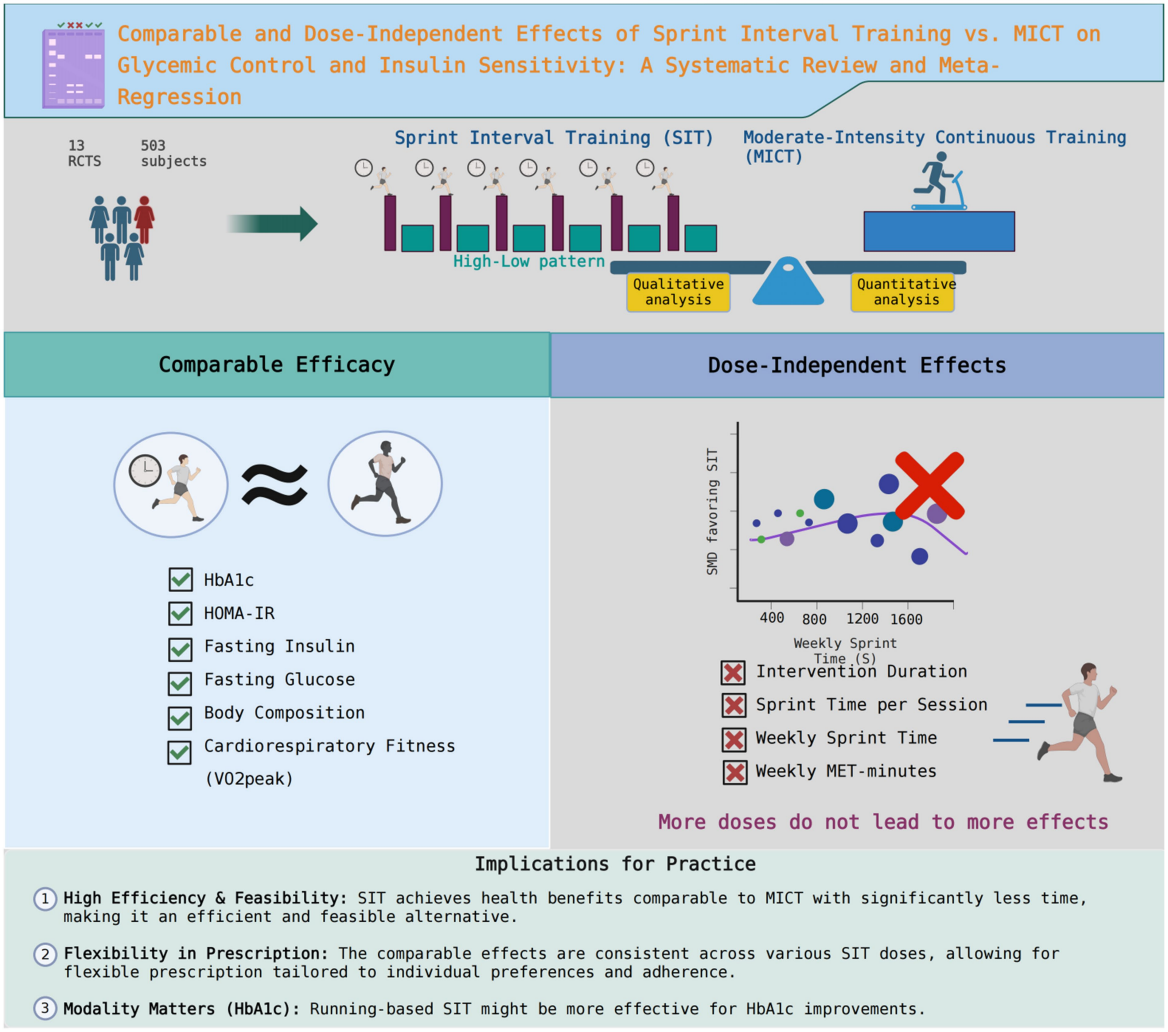
Graphical summary of the systematic review and meta-analysis. The figure illustrates the study’s key outcomes. Top: The research question compares sprint interval training (SIT) and moderate-intensity continuous training (MICT) for adults with metabolic dysfunction. Middle: The study methodology included a meta-analysis of 13 randomized controlled trials (RCTs) with 503 participants. Bottom: The main comparative findings highlighted outcomes where SIT was superior to MICT (decreased fasting insulin and increased M-value) and those where their effects were equivalent. The conditional superiority of SIT for improving glycated hemoglobin (HbA1c), which requires running-based exercise for ≥8 weeks, is also detailed. SIT, sprint interval training; MICT, moderate-intensity continuous training; RCTs, randomized controlled trials; HbA1c, glycated hemoglobin.

Crucially, our series of univariable meta-regression analyses found no significant dose–response relationships for any of the investigated outcomes. Variations in key training parameters—including total intervention duration, per-session sprint volume, total weekly sprint time, or cumulative weekly physiological load (MET-minutes)—did not significantly alter the comparative effectiveness of SIT. However, our subgroup analyses suggest that interventions utilizing a running modality or lasting 8 weeks or longer may be more effective at improving HbA1c, highlighting potential moderating factors beyond training dose alone. The main findings of this study are summarized in the graphical abstract (Figure 8).

### 4.2 Potential mechanisms

The consistent finding of comparable outcomes despite vastly different time commitments underscores the unique physiological stimulus provided by SIT. A plausible, though speculative, explanation is that the high-intensity nature of SIT preferentially recruits type II muscle fibers (38), thereby inducing a more pronounced and rapid enhancement of glucose transport machinery (e.g., glucose transporter 4 [GLUT4] translocation) than MICT (39). In contrast, HbA1c, which reflects average glycemia over several months, may be more strongly influenced by total energy expenditure and sustained metabolic adaptations, factors that could be comparable between well-matched SIT and MICT protocols.

Mechanistically, it is thought that the supramaximal intensity of SIT necessitates near-maximal activation of the entire motor unit pool, including the glycolytic type II muscle fibers that are often undertrained in conventional endurance exercise (38). This extensive fiber recruitment leads to a rapid and substantial depletion of intramuscular fuel reserves, particularly phosphocreatine and glycogen (6). This metabolic stress is a potent stimulus for activating key signaling kinases, most notably AMP-activated protein kinase (AMPK) (40). Activated AMPK, acting as a cellular energy sensor, initiates a cascade of downstream events that, in the long term, leads to mitochondrial biogenesis, primarily via the master regulator PGC-1α (41). On an acute level, this signaling cascade also promotes insulin-independent translocation of GLUT4 glucose transporters to the cell surface, enhancing muscle glucose uptake (39). The long-term repetition of SIT leads to an increase in total GLUT4 protein content, which, combined with improved insulin signaling pathway function (e.g., enhanced Akt phosphorylation), contributes to the durable improvements in insulin sensitivity observed in our analysis (40, 41).

### 4.3 Public health and practical implications

Our meta-analysis paints a more nuanced picture of the relative efficacy between SIT and MICT. The finding of comparable effects across many cardiometabolic outcomes is, in itself, profound in terms of time efficiency. Across the included studies, SIT protocols required up to 60–75% less total exercise time to achieve similar health outcomes as MICT. This is consistent with seminal work and subsequent research (5, 38), confirming that SIT can induce physiological adaptations comparable to MICT with a fraction of the time commitment. This is a critical consideration in clinical and public health contexts where “lack of time” remains a primary barrier to exercise participation (42, 43).

While both forms of exercise improve metabolic health, the unique intensity profile of SIT may confer specific advantages in enhancing peripheral insulin action. Conversely, both training modes appear similarly effective in improving blood pressure and cardiorespiratory fitness. While both forms of exercise improve metabolic health, the unique intensity profile of SIT may confer specific advantages in enhancing peripheral insulin action. Conversely, both training modes appear similarly effective in improving blood pressure and cardiorespiratory fitness.

Adherence is key to the long-term success of any exercise intervention. Contrary to the common assumption that the extreme intensity and associated discomfort of SIT might compromise enjoyment and adherence, our systematic review does not support this notion. The seven trials that reported this metric showed very high adherence rates for SIT (78–100%), comparable to those observed in MICT groups (>80–100%). This may be because while SIT involves higher subjective fatigue during the session, the post-exercise affective response and enjoyment can be comparable to MICT after an adaptation period (44, 45). This indicates that SIT is not only an effective and time-efficient option but also a surprisingly sustainable mode of exercise for many individuals with metabolic dysfunction.

Our findings provide important and actionable insights for clinical and public health practice.

(1) SIT as a first-line or alternative exercise prescription: Given that a perceived “lack of time” is the primary barrier to exercise adherence for most adults (42, 43). Our analysis provides convincing evidence for clinicians and public health practitioners to confidently recommend SIT as a primary exercise option. Its effects are comparable to MICT across many outcomes, yet it requires significantly less time and demonstrates high adherence (46). For sedentary individuals with metabolic dysfunction who struggle to meet the standard 150-min weekly aerobic guideline, 2–3 brief SIT sessions per week represent a feasible and evidence-based starting point to improve glycemic control and insulin sensitivity, a conclusion supported by recent expert consensus statements (47).
(2) Patient-centered shared decision-making: The choice between SIT and MICT should be guided by patient preference and individual circumstances. Our data, showing high adherence rates for supervised SIT, suggest that intensity is not an insurmountable barrier for motivated individuals, possibly due to the motivating nature of its short, challenging structure. Our findings support a shared decision-making process in which practitioners present SIT not as a lesser “shortcut” but as a physiologically equivalent stimulus (48). Moreover, supervised settings provide a controlled environment for learning correct technique, thus reducing injury risk (49), while the encouragement and accountability from professionals are critical for maintaining motivation (50).
(3) Emphasis on regularity over total volume: The finding that even short-term SIT protocols (e.g., 2 weeks) can yield metabolic benefits underscores the importance of ‘regularity’. For sedentary individuals, the primary goal should be to establish a consistent exercise habit. The low time commitment per session of SIT makes integrating regular physical activity into a busy schedule more feasible. The practical focus should be on cultivating the habit of regular, intense physical exertion, as regularity, rather than total volume, is often the key determinant of long-term health benefits (51).
(4) The Quality of Stimulus May Matter More Than Quantity: The lack of a dose–response relationship is a clinically important finding. It suggests that practitioners do not necessarily need to prescribe higher volumes of SIT to achieve better results. The key may be achieving the maximal-intensity stimulus inherent in SIT, which appears to trigger potent physiological adaptations even with minimal volume. This simplifies the exercise prescription and can improve patient confidence and adherence.

### 4.4 Limitations and future directions

This meta-analysis has several strengths, including a comprehensive search strategy, a pre-registered protocol, strict inclusion of only RCTs, and rigorous analytical methods, such as detailed subgroup analyses and GRADE ratings. The parallel analysis structure clearly distinguishes the effects of SIT against both non-exercise and active (MICT) controls. Nevertheless, several limitations must be acknowledged. First, there is significant heterogeneity in the SIT protocols themselves (e.g., sprint duration, repetitions, recovery modes), a common challenge in the field (52, 53). Second, the relatively short duration of most included trials (≤12 weeks) limits inferences about the long-term effects of SIT. Third, while we included studies that used gold standard methods, many trials still rely on surrogate markers, such as HOMA-IR. Fourth, our finding of no dose–response relationship should be interpreted with caution. It is possible this reflects a limited range and variability of the training doses in the included studies rather than a true biological dose-independence, and our analysis may have been underpowered to detect subtle dose-effects. Fifth, our review primarily included middle-aged adults, and our results may not be generalizable to older adults (>65 years), who were underrepresented. Sixth, our search was limited to peer-reviewed articles and did not include gray literature, which could introduce a potential publication bias, although Egger’s test did not suggest this was a major issue. Finally, while a major strength of this study was the inclusion of several dose–response meta-regression analyses, these analyses were ultimately limited by the small number of studies available for each outcome. Therefore, the consistent null findings should be interpreted as demonstrating no detectable dose–response relationship within the available evidence, while acknowledging that the statistical power to detect subtle trends was limited.

While consolidating existing evidence, this meta-analysis also illuminates several critical knowledge gaps.

(1) Long-term adherence and sustainability: Most included trials were relatively short (≤16 weeks). A crucial unresolved question is whether individuals can adhere to SIT protocols over the long term (≥1 year) in unsupervised, real-world settings. Future studies should employ hybrid effectiveness-implementation designs to not only assess long-term health outcomes but also to identify the behavioral and psychological factors that predict long-term SIT adherence beyond the supervised laboratory environment (54).
(2) Identifying the Minimal Effective Dose: Our analysis showed that even low-volume SIT was effective. A crucial next step is to design studies specifically aimed at identifying the minimal effective dose of SIT required to elicit meaningful improvements in glycemic control. Answering this is key to developing the most efficient and sustainable exercise prescriptions possible.
(3) Exploring modality-specific effects: Our subgroup analysis hinted that running-based SIT may be more effective than cycling for improving HbA1c. This is a novel and potentially significant finding that requires dedicated mechanistic validation. Future research could utilize advanced imaging and biopsy techniques to compare the muscle mass and fiber types recruited by different SIT modalities and link these findings to effects on whole-body glucose homeostasis, as the total muscle mass activated is a key determinant of the metabolic impact of exercise (55, 56).
(4) Focusing on underrepresented populations: Although our review included mixed-sex studies, research on the effects of SIT in specific populations remains lacking. For instance, postmenopausal women, who experience unique hormonal shifts affecting metabolic health, may respond differently to SIT. Similarly, more research is needed in older adults (>65 years). While HIIT is safe and effective in older populations (57, 58), more SIT-specific data are required to establish its unique risk–benefit profile in this growing demographic.
(5) Moving beyond glycemic control: Future research should broaden the scope of outcome measures. Investigating the effects of SIT on novel biomarkers of cardiometabolic health, such as systemic inflammation (e.g., high-sensitivity C-reactive protein [hs-CRP]), endothelial function, and ectopic fat storage (e.g., liver and pancreas fat via magnetic resonance imaging [MRI]), would provide a more complete understanding of its systemic benefits and its role in multi-organ health (59).

## 5 Conclusion

Our results indicate that Sprint Interval Training (SIT) elicits cardiometabolic improvements comparable to moderate-intensity continuous training (MICT) in adults with metabolic dysfunction. This suggests that SIT is a viable and time-efficient alternative to MICT for individuals with limited time availability. A key finding was that, within the dose range included in the analyzed studies, this equivalence appeared independent of training dose; however, this should be interpreted with caution, as it may be limited by statistical power and protocol variability in the available literature.

From a public health perspective, these findings support SIT as a powerful tool for practitioners to prescribe and for individuals to adopt, particularly when time commitment is a significant barrier to regular physical activity. However, variability in SIT protocols and moderate statistical heterogeneity warrant cautious interpretation and emphasize the need for patient-centered shared decision-making.

## Data Availability

The raw data supporting the conclusions of this article will be made available by the authors, without undue reservation.
