# Supplementary material for: A time-efficient public health strategy: a systematic review and meta-regression on the comparable and dose-independent effects of sprint interval training vs. moderate-intensity continuous training for metabolic health
Source: Front Public Health. 2026 Jan 6;13:1708893. doi: 10.3389/fpubh.2025.1708893 (PMC12816250; doi:10.3389/fpubh.2025.1708893)
Supplement: Supplementary file 1 [file Data_Sheet_1.docx]

Supplementary Material

Table S1. Search Strategy.

Table S2. Summary of subgroup analyses for primary and secondary outcomes.

Table S3. PRISMA 2020 Checklist.

Table S4. Meta-Regression Analyses for Dose-Response Relationships on Glycemic Outcomes.

Figure S1. Risk of bias summary.

Figure S2. Risk of bias traffic light plot.

Figures S3-S18. Leave-one-out sensitivity analyses.

Figures S19-S31. Funnel plots for the assessment of publication bias.

# Table S1. Search Strategy.

Pubmed

| #1 | "sprint interval training"[Title/Abstract] OR "sprint interval exercise"[Title/Abstract] OR "sprint intermittent training"[Title/Abstract] OR "sprint training"[Title/Abstract] OR "sprint-interval training"[Title/Abstract] OR "High-Intensity Interval Training"[Mesh] OR "High intensity intermittent training"[Title/Abstract] OR "High-intensity intermittent training"[Title/Abstract] OR "Interval training"[Title/Abstract] OR "interval exercise"[Title/Abstract] OR "HIIT"[Title/Abstract] OR "high intensity exercise"[Title/Abstract] OR "high intensity aerobic interval training"[Title/Abstract] OR "all-out exercise training"[Title/Abstract] OR "all-out training"[Title/Abstract] OR "all-out interval training"[Title/Abstract] OR "Supramaximal interval training"[Title/Abstract] OR "Wingate training"[Title/Abstract] OR "maximal intensity"[Title/Abstract] |
| --- | --- |
| #2 | "Diabetes Mellitus, Type 2"[Mesh] OR "Prediabetic State"[Mesh] OR "Insulin Resistance"[Mesh] OR "Metabolic Syndrome"[Mesh] OR "type 2 diabetes"[tiab] OR "T2DM"[tiab] OR "prediabetes"[tiab] OR "metabolic syndrome"[tiab] OR "insulin resistance"[tiab] OR "glycemic control"[tiab] OR "glucose tolerance"[tiab] OR overweight[tiab] OR obese[tiab] ) |
| #3 | "randomized controlled trial"[pt] OR "controlled clinical trial"[pt] OR randomized[tiab] OR placebo[tiab] OR "drug therapy"[sh] OR randomly[tiab] OR trial[tiab] OR groups[tiab] |
| #4 | #1 AND #2 AND #3 |

Web of Science

| #1 | TS=("sprint interval training" OR "sprint interval exercise" OR "Sprint intermittent training" OR "sprint training" OR "sprint-interval training" OR "High-Intensity Interval Training" OR "High intensity intermittent training" OR "High-intensity intermittent training" OR "Interval training" OR "interval exercise" OR "HIIT" OR "high intensity exercise" OR "high intensity aerobic interval training" OR "all-out exercise training" OR "all-out training" OR "all-out interval training" OR "Supramaximal interval training" OR "Wingate training" OR "maximal intensity") |
| --- | --- |
| #2 | TS=("type 2 diabetes" OR T2DM OR prediabetes OR "metabolic syndrome" OR "insulin resistance" OR "glycemic control" OR "glucose tolerance" OR overweight OR obese) |
| #3 | TS=(randomized OR randomised OR placebo OR "controlled trial" OR trial OR groups) |
| #4 | #1 AND #2 AND #3 |

SPORTDiscus

| S1 | DE "HIGH intensity interval training" OR DE "SPRINT training" OR DE "INTERVAL training" OR TI ( "sprint interval training" OR "sprint interval exercise" OR "Sprint intermittent training" OR "sprint training" OR "sprint-interval training" OR "High-Intensity Interval Training" OR "High intensity intermittent training" OR "High-intensity intermittent training" OR "Interval training" OR "interval exercise" OR "HIIT" OR "high intensity exercise" OR "high intensity aerobic interval training" OR "all-out exercise training" OR "all-out training" OR "all-out interval training" OR "Supramaximal interval training" OR "Wingate training" OR "maximal intensity" ) OR AB ( "sprint interval training" OR "sprint interval exercise" OR "Sprint intermittent training" OR "sprint training" OR "sprint-interval training" OR "High-Intensity Interval Training" OR "High intensity intermittent training" OR "High-intensity intermittent training" OR "Interval training" OR "interval exercise" OR "HIIT" OR "high intensity exercise" OR "high intensity aerobic interval training" OR "all-out exercise training" OR "all-out training" OR "all-out interval training" OR "Supramaximal interval training" OR "Wingate training" OR "maximal intensity" ) |
| --- | --- |
| S2 | DE "TYPE II diabetes" OR DE "PREDIABETES" OR DE "INSULIN resistance" OR DE "METABOLIC syndrome" OR TI ( "type 2 diabetes" OR T2DM OR prediabetes OR "insulin resistance" OR "glycemic control" OR overweight OR obese ) OR AB ( "type 2 diabetes" OR T2DM OR prediabetes OR "insulin resistance" OR "glycemic control" OR overweight OR obese ) |
| S3 | TI ( randomized OR randomised OR placebo OR "controlled trial" OR trial OR groups ) OR AB ( randomized OR randomised OR placebo OR "controlled trial" OR trial OR groups ) |
| S4 | S1 AND S2 AND S3 |

Cochrane Library

| #1 | [mh "High-Intensity Interval Training"] OR "sprint interval training":ti,ab,kw OR "sprint interval exercise":ti,ab,kw OR "Sprint intermittent training":ti,ab,kw OR "sprint training":ti,ab,kw OR "sprint-interval training":ti,ab,kw OR "High intensity intermittent training":ti,ab,kw OR "High-intensity intermittent training":ti,ab,kw OR "Interval training":ti,ab,kw OR "interval exercise":ti,ab,kw OR "HIIT":ti,ab,kw OR "high intensity exercise":ti,ab,kw OR "high intensity aerobic interval training":ti,ab,kw OR "all-out exercise training":ti,ab,kw OR "all-out training":ti,ab,kw OR "all-out interval training":ti,ab,kw OR "Supramaximal interval training":ti,ab,kw OR "Wingate training":ti,ab,kw OR "maximal intensity":ti,ab,kw |
| --- | --- |
| #2 | [mh "Diabetes Mellitus, Type 2"] OR [mh "Prediabetic State"] OR [mh "Insulin Resistance"] OR [mh "Metabolic Syndrome"] |
| #3 | #1 AND #2 |

Embase

| #1 | 'high intensity interval training'/exp OR 'sprint interval training':ti,ab,kw OR 'sprint interval exercise':ti,ab,kw OR 'sprint intermittent training':ti,ab,kw OR 'sprint training':ti,ab,kw OR 'sprint-interval training':ti,ab,kw OR 'high intensity intermittent training':ti,ab,kw OR 'high-intensity intermittent training':ti,ab,kw OR 'interval training':ti,ab,kw OR 'interval exercise':ti,ab,kw OR 'hiit':ti,ab,kw OR 'high intensity exercise':ti,ab,kw OR 'high intensity aerobic interval training':ti,ab,kw OR 'all-out exercise training':ti,ab,kw OR 'all-out training':ti,ab,kw OR 'all-out interval training':ti,ab,kw OR 'supramaximal interval training':ti,ab,kw OR 'wingate training':ti,ab,kw OR 'maximal intensity':ti,ab,kw |
| --- | --- |
| #2 | 'type 2 diabetes mellitus'/exp OR 'prediabetes'/exp OR 'insulin resistance'/exp OR 'metabolic syndrome'/exp OR 'type 2 diabetes':ti,ab OR 'T2DM':ti,ab OR 'prediabetes':ti,ab OR 'metabolic syndrome':ti,ab OR 'insulin resistance':ti,ab OR 'glycemic control':ti,ab OR 'glucose tolerance':ti,ab OR 'overweight':ti,ab OR 'obese':ti,ab |
| #3 | 'randomized controlled trial'/exp OR 'controlled clinical trial'/exp OR 'randomization'/exp OR 'placebo'/exp OR 'randomly':ti,ab OR 'trial':ti,ab OR 'groups':ti,ab |
| #4 | #1 AND #2 AND #3 |

Scopus

|  | TITLE-ABS-KEY ( "sprint interval training" OR "sprint interval exercise" OR "Sprint intermittent training" OR "sprint training" OR "sprint-interval training" OR "High-Intensity Interval Training" OR "High intensity intermittent training" OR "High-intensity intermittent training" OR "Interval training" OR "interval exercise" OR "HIIT" OR "high intensity exercise" OR "high intensity aerobic interval training" OR "all-out exercise training" OR "all-out training" OR "all-out interval training" OR "Supramaximal interval training" OR "Wingate training" OR "maximal intensity" ) |
| --- | --- |
| AND |  |
|  | TITLE-ABS-KEY("type 2 diabetes" OR T2DM OR prediabetes OR "metabolic syndrome" OR "insulin resistance" OR "glycemic control" OR "glucose tolerance" OR overweight OR obese) |
| AND |  |
|  | TITLE-ABS-KEY(randomized OR randomised OR placebo OR "controlled trial" OR trial OR groups) |

# Table S2. Summary of subgroup analyses for primary and secondary outcomes.

| **Outcomes** | **Subgroups** | **No.**  **Studies** | **N** | **Meta-analyses** |  | **Heterogeneity** |  | **Moderation**  **effect** |
| --- | --- | --- | --- | --- | --- | --- | --- | --- |
|  |  |  |  | **MD(95%CI)** | **p** | **l²** | **p** | **p** |
| **HbAlc** | **Healthy Status** |  |  |  |  |  |  |  |
|  | Diabetic/Prediabetic | 5 | 142 | -0.02 (-0.11, 0.07) | > 0.05 | 22.80% | 0.271 | 0.767 |
|  | Overweight/At-Risk | 1 | 12 | 0.02 (-0.25, 0.29) | > 0.05 | N/A | N/A |  |
|  | **Age** |  |  |  |  |  |  |  |
|  | ≥ 45 years | 4 | 68 | 0.03 (-0.06, 0.12) | > 0.05 | 0.00% | 0.657 | 0.082 |
|  | < 45 years | 2 | 86 | -0.09 (-0.18, 0.01) | > 0.05 | 0.00% | 0.417 |  |
|  | **modality** |  |  |  |  |  |  |  |
|  | Cycling | 4 | 63 | 0.04 (-0.05, 0.12) | > 0.05 | 0.00% | 0.788 | 0.040 |
|  | Running | 2 | 91 | -0.10 (-0.19, -0.01) | < 0.05 | 0.00% | 0.953 |  |
|  | **Sex** |  |  |  |  |  |  |  |
|  | male | 2 | 86 | -0.09 (-0.18, 0.01) | > 0.05 | 0.00% | 0.417 | 0.082 |
|  | mixed | 4 | 68 | 0.03 (-0.06, 0.12) | > 0.05 | 0.00% | 0.657 |  |
|  | **Duration** |  |  |  |  |  |  |  |
|  | ≥ 8 weeks | 2 | 91 | -0.10 (-0.19, -0.01) | < 0.05 | 0.00% | 0.953 | 0.040 |
|  | < 8 weeks | 4 | 63 | -0.04 (-0.05, 0.12) | > 0.05 | 0.00% | 0.788 |  |
| **HOMA-IR** | **Healthy Status** |  |  |  |  |  |  |  |
|  | Diabetic/Prediabetic | 6 | 156 | -0.18 (-0.37, 0.01) | > 0.05 | 0.00% | 0.847 | 0.190 |
|  | Overweight/At-Risk | 2 | 22 | 0.11 (-0.28, 0.49) | > 0.05 | 46.50% | 0.171 |  |
|  | **Age** |  |  |  |  |  |  |  |
|  | ≥ 45 years | 5 | 82 | -0.17 (-0.40, 0.05) | > 0.05 | 0.00% | 0.736 | 0.385 |
|  | < 45 years | 3 | 96 | 0.00 (-0.31, 0.32) | > 0.05 | 50.60% | 0.132 |  |
|  | **modality** |  |  |  |  |  |  |  |
|  | Cycling | 6 | 87 | -0.05 (-0.31, 0.20) | > 0.05 | 27.10% | 0.231 | 0.562 |
|  | Running | 2 | 91 | -0.17 (-0.46, 0.13) | > 0.05 | 0.00% | 0.774 |  |
|  | **Sex** |  |  |  |  |  |  |  |
|  | male | 3 | 98 | 0.06 (-0.39, 0.50) | > 0.05 | 45.90% | 0.158 | 0.668 |
|  | female | 1 | 14 | -0.13 (-0.60, 0.34) | > 0.05 | N/A | N/A |  |
|  | mixed | 4 | 66 | -0.17 (-0.40, 0.05) | > 0.05 | 0.00% | 0.633 |  |
|  | **Duration** |  |  |  |  |  |  |  |
|  | ≥ 8 weeks | 4 | 121 | -0.15 (-0.40, 0.10) | > 0.05 | 0.00% | 0.946 | 0.615 |
|  | < 8 weeks | 4 | 57 | -0.05 (-0.37, 0.27) | > 0.05 | 53.60% | 0.091 |  |
| **Fasting glucose** | **Healthy Status** |  |  |  |  |  |  |  |
|  | Diabetic/Prediabetic | 6 | 156 | -0.09 (-0.27, 0.09) | > 0.05 | 0.00% | 0.477 | 0.158 |
|  | Overweight/At-Risk | 3 | 34 | 0.18 (-0.15, 0.51) | > 0.05 | 41.00% | 0.184 |  |
|  | **Age** |  |  |  |  |  |  |  |
|  | ≥ 45 years | 6 | 82 | -0.09 (-0.35, 0.17) | > 0.05 | 0.00% | 0.411 | 0.295 |
|  | < 45 years | 4 | 108 | 0.09 (-0.06, 0.23) | > 0.05 | 47.80% | 0.124 |  |
|  | **modality** |  |  |  |  |  |  |  |
|  | Cycling | 7 | 99 | 0.04 (-0.21, 0.29) | > 0.05 | 54.40% | 0.041 | 0.706 |
|  | Running | 2 | 91 | -0.02 (-0.22, 0.18) | > 0.05 | 0.00% | 0.654 |  |
|  | **Sex** |  |  |  |  |  |  |  |
|  | male | 3 | 98 | -0.02 (-0.23, 0.20) | > 0.05 | 0.00% | 0.615 | 0.028 |
|  | female | 1 | 14 | 0.33 (0.07, 0.59) | < 0.05 | N/A | N/A |  |
|  | mixed | 6 | 78 | -0.10 (-0.30, 0.09) | > 0.05 | 0.00% | 0.425 |  |
|  | **Duration** |  |  |  |  |  |  |  |
|  | ≥ 8 weeks | 5 | 133 | 0.09 (-0.06, 0.23) | > 0.05 | 27.90% | 0.235 | 0.287 |
|  | < 8 weeks | 4 | 57 | -0.11 (-0.41, 0.19) | > 0.05 | 15.80% | 0.313 |  |
| **Fasting insulin** | **Healthy Status** |  |  |  |  |  |  |  |
|  | Diabetic/Prediabetic | 6 | 156 | -0.46 (-1.42, 0.51) | > 0.05 | 0.00% | 0.931 | 0.938 |
|  | Overweight/At-Risk | 2 | 22 | -0.53 (-2.15, 1.09) | > 0.05 | 0.00% | 0.651 |  |
|  | **Age** |  |  |  |  |  |  |  |
|  | ≥ 45 years | 5 | 82 | -0.74 (-2.13, 0.66) | > 0.05 | 0.00% | 0.903 | 0.648 |
|  | < 45 years | 3 | 96 | -0.33 (-1.36, 0.70) | > 0.05 | 0.00% | 0.861 |  |
|  | **modality** |  |  |  |  |  |  |  |
|  | Cycling | 6 | 87 | -0.64 (-1.74, 0.47) | > 0.05 | 0.00% | 0.937 | 0.667 |
|  | Running | 2 | 91 | -0.27 (-1.51, 0.97) | > 0.05 | 0.00% | 0.768 |  |
|  | **Sex** |  |  |  |  |  |  |  |
|  | male | 3 | 98 | -0.23 (-1.43, 0.96) | > 0.05 | 0.00% | 0.674 | 0.858 |
|  | female | 1 | 14 | -0.78 (-2.73, 1.17) | > 0.05 | N/A | N/A |  |
|  | mixed | 4 | 66 | -0.65 (-2.06, 0.77) | > 0.05 | 0.00% | 0.929 |  |
|  | **Duration** |  |  |  |  |  |  |  |
|  | ≥ 8 weeks | 4 | 121 | -0.47 (-1.51, 0.57) | > 0.05 | 0.00% | 0.813 | 0.990 |
|  | < 8 weeks | 4 | 57 | -0.48 (-1.84, 0.88) | > 0.05 | 0.00% | 0.896 |  |
| **VO₂peak** | **Healthy Status** |  |  |  |  |  |  |  |
|  | Diabetic/Prediabetic | 5 | 84 | 0.94 (-0.66, 2.53) | > 0.05 | 0.00% | 0.908 | 0.365 |
|  | Overweight/At-Risk | 4 | 46 | -0.23 (-2.20, 1.74) | > 0.05 | 0.00% | 0.699 |  |
|  | **Age** |  |  |  |  |  |  |  |
|  | ≥ 45 years | 5 | 84 | 0.94 (-0.66, 2.53) | > 0.05 | 0.00% | 0.908 | 0.365 |
|  | < 45 years | 4 | 46 | -0.23 (-2.20, 1.74) | > 0.05 | 0.00% | 0.699 |  |
|  | **modality** |  |  |  |  |  |  |  |
|  | Cycling | 8 | 113 | 0.60 (-0.68, 1.89) | > 0.05 | 0.00% | 0.910 | 0.463 |
|  | Running | 1 | 17 | -1.20 (-5.84, 3.44) | > 0.05 | N/A | N/A |  |
|  | **Sex** |  |  |  |  |  |  |  |
|  | male | 3 | 36 | -0.29 (-2.63, 2.06) | > 0.05 | 0.00% | 0.526 | 0.627 |
|  | female | 1 | 14 | -0.10 (-3.25, 3.05) | > 0.05 | N/A | N/A |  |
|  | mixed | 5 | 80 | 1.01 (-0.64, 2.66) | > 0.05 | 0.00% | 0.904 |  |
|  | **Duration** |  |  |  |  |  |  |  |
|  | ≥ 8 weeks | 4 | 59 | 0.34 (-1.47, 2.15) | > 0.05 | 0.00% | 0.838 | 0.842 |
|  | < 8 weeks | 5 | 71 | 0.59 (-1.10, 2.29) | > 0.05 | 0.00% | 0.668 |  |
| **SBP** | **Healthy Status** |  |  |  |  |  |  |  |
|  | Diabetic/Prediabetic | 2 | 33 | -3.91 (-11.48, 3.66) | > 0.05 | 0.00% | 0.728 | 0.711 |
|  | Overweight/At-Risk | 3 | 32 | -2.01 (-8.61, 4.60) | > 0.05 | 0.00% | 0.760 |  |
|  | **Age** |  |  |  |  |  |  |  |
|  | ≥ 45 years | 2 | 33 | -3.91 (-11.48, 3.66) | > 0.05 | 0.00% | 0.728 | 0.711 |
|  | < 45 years | 3 | 32 | -2.01 (-8.61, 4.60) | > 0.05 | 0.00% | 0.760 |  |
|  | **modality** |  |  |  |  |  |  |  |
|  | Cycling | 4 | 48 | -2.35 (-7.70, 3.01) | > 0.05 | 0.00% | 0.902 | 0.632 |
|  | Running | 1 | 17 | -5.90 (-19.42, 7.62) | > 0.05 | N/A | N/A |  |
|  | **Sex** |  |  |  |  |  |  |  |
|  | male | 3 | 36 | -1.33 (-7.88, 5.22) | > 0.05 | 0.00% | 0.861 | 0.490 |
|  | female |  |  |  |  |  |  |  |
|  | mixed | 2 | 29 | -4.88 (-12.55, 2.78) | > 0.05 | 0.00% | 0.858 |  |
|  | **Duration** |  |  |  |  |  |  |  |
|  | ≥ 8 weeks | 3 | 45 | -4.11 (-9.98, 1.77) | > 0.05 | 0.00% | 0.938 | 0.422 |
|  | < 8 weeks | 2 | 20 | 0.43 (-8.96, 9.81) | > 0.05 | 0.00% | 0.850 |  |
| **DBP** | **Healthy Status** |  |  |  |  |  |  |  |
|  | Diabetic/Prediabetic | 2 | 33 | -0.07 (-4.16, 4.01) | > 0.05 | 0.00% | 0.528 | 0.748 |
|  | Overweight/At-Risk | 3 | 32 | 0.87 (-3.18, 4.92) | > 0.05 | 0.00% | 0.590 |  |
|  | **Age** |  |  |  |  |  |  |  |
|  | ≥ 45 years | 2 | 33 | -0.07 (-4.16, 4.01) | > 0.05 | 0.00% | 0.528 | 0.748 |
|  | < 45 years | 3 | 32 | 0.87 (-3.18, 4.92) | > 0.05 | 0.00% | 0.590 |  |
|  | **modality** |  |  |  |  |  |  |  |
|  | Cycling | 4 | 48 | 0.13 (-3.02, 3.27) | > 0.05 | 0.00% | 0.711 | 0.673 |
|  | Running | 1 | 17 | 1.80 (-5.30, 8.90) | > 0.05 | N/A | N/A |  |
|  | **Sex** |  |  |  |  |  |  |  |
|  | male | 3 | 36 | 0.31 (-3.62, 4.24) | > 0.05 | 0.00% | 0.508 | 0.947 |
|  | female |  |  |  |  |  |  |  |
|  | mixed | 2 | 29 | 0.51 (-3.72, 4.73) | > 0.05 | 0.00% | 0.657 |  |
|  | **Duration** |  |  |  |  |  |  |  |
|  | ≥ 8 weeks | 3 | 45 | -0.12 (-3.35, 3.11) | > 0.05 | 0.00% | 0.819 | 0.483 |
|  | < 8 weeks | 2 | 20 | 2.43 (-3.92, 8.77) | > 0.05 | 0.00% | 0.415 |  |
| **Body Fat** | **Healthy Status** |  |  |  |  |  |  |  |
|  | Diabetic/Prediabetic | 4 | 65 | 0.07 (-2.00, 2.14) | > 0.05 | 0.00% | 0.998 | 0.898 |
|  | Overweight/At-Risk | 3 | 32 | -0.13 (-2.42, 2.16) | > 0.05 | 0.00% | 0.853 |  |
|  | **Age** |  |  |  |  |  |  |  |
|  | ≥ 45 years | 4 | 65 | 0.07 (-2.00, 2.14) | > 0.05 | 0.00% | 0.998 | 0.898 |
|  | < 45 years | 3 | 32 | -0.13 (-2.42, 2.16) | > 0.05 | 0.00% | 0.853 |  |
|  | **Sex** |  |  |  |  |  |  |  |
|  | male | 3 | 36 | 0.06 (-2.02, 2.15) | > 0.05 | 0.00% | 0.834 | 0.908 |
|  | female |  |  |  |  |  |  |  |
|  | mixed | 4 | 61 | -0.12 (-2.39, 2.15) | > 0.05 | 0.00% | 1.000 |  |
|  | **Duration** |  |  |  |  |  |  |  |
|  | ≥ 8 weeks | 2 | 28 | 0.14 (-2.23, 2.52) | > 0.05 | 0.00% | 0.872 | 0.858 |
|  | < 8 weeks | 5 | 69 | -0.14 (-2.16, 1.88) | > 0.05 | 0.00% | 0.988 |  |
| **Triglycerides** | **Healthy Status** |  |  |  |  |  |  |  |
|  | Diabetic/Prediabetic | 4 | 65 | 0.03 (-0.19, 0.24) | > 0.05 | 0.00% | 0.771 | 0.729 |
|  | Overweight/At-Risk | 2 | 24 | -0.37 (-2.52, 1.79) | > 0.05 | 80.40% | 0.024 |  |
|  | **Age** |  |  |  |  |  |  |  |
|  | ≥ 45 years | 4 | 65 | 0.03 (-0.19, 0.24) | > 0.05 | 0.00% | 0.771 | 0.729 |
|  | < 45 years | 2 | 24 | -0.37 (-2.52, 1.79) | > 0.05 | 80.40% | 0.024 |  |
|  | **Sex** |  |  |  |  |  |  |  |
|  | male | 2 | 28 | 0.10 (-0.40, 0.60) | > 0.05 | 0.00% | 0.325 | 0.457 |
|  | female |  |  |  |  |  |  |  |
|  | mixed | 4 | 61 | -0.14 (-0.52, 0.24) | > 0.05 | 48.20% | 0.122 |  |
|  | **Duration** |  |  |  |  |  |  |  |
|  | ≥ 8 weeks | 2 | 28 | -0.60 (-2.01, 0.81) | > 0.05 | 74.80% | 0.046 | 0.373 |
|  | < 8 weeks | 4 | 61 | 0.05 (-0.18, 0.28) | > 0.05 | 0.00% | 0.548 |  |
| **HDL-C** | **Healthy Status** |  |  |  |  |  |  |  |
|  | Diabetic/Prediabetic | 4 | 65 | -0.02 (-0.15, 0.12) | > 0.05 | 22.10% | 0.278 | 0.571 |
|  | Overweight/At-Risk | 2 | 24 | 0.03 (-0.07, 0.14) | > 0.05 | 0.00% | 0.939 |  |
|  | **Age** |  |  |  |  |  |  |  |
|  | ≥ 45 years | 4 | 65 | -0.02 (-0.15, 0.12) | > 0.05 | 22.10% | 0.278 | 0.571 |
|  | < 45 years | 2 | 24 | 0.03 (-0.07, 0.14) | > 0.05 | 0.00% | 0.939 |  |
|  | **Sex** |  |  |  |  |  |  |  |
|  | male | 2 | 28 | 0.08 (-0.03, 0.20) | > 0.05 | 0.00% | 0.661 | 0.160 |
|  | female |  |  |  |  |  |  |  |
|  | mixed | 4 | 61 | -0.03 (-0.12, 0.07) | > 0.05 | 0.00% | 0.597 |  |
|  | **Duration** |  |  |  |  |  |  |  |
|  | ≥ 8 weeks | 2 | 28 | 0.06 (-0.03, 0.15) | > 0.05 | 0.00% | 0.450 | 0.121 |
|  | < 8 weeks | 4 | 61 | -0.06 (-0.18, 0.06) | > 0.05 | 0.00% | 0.795 |  |

p value for moderation effect indicates the statistical significance of the difference in effect between subgroups. Abbreviations: MD, mean difference; CI, confidence interval; N/A, not applicable.

#
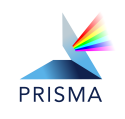
Table S3. PRISMA 2020 Checklist.

| **Section and Topic** | **Item #** | **Checklist item** | **Location where item is reported** |
| --- | --- | --- | --- |
| **TITLE** | | |  |
| Title | 1 | Identify the report as a systematic review. | 1 |
| **ABSTRACT** | | |  |
| Abstract | 2 | See the PRISMA 2020 for Abstracts checklist. | 1-2 |
| **INTRODUCTION** | | |  |
| Rationale | 3 | Describe the rationale for the review in the context of existing knowledge. | 2 |
| Objectives | 4 | Provide an explicit statement of the objective(s) or question(s) the review addresses. | 2-3 |
| **METHODS** | | |  |
| Eligibility criteria | 5 | Specify the inclusion and exclusion criteria for the review and how studies were grouped for the syntheses. | 3 |
| Information sources | 6 | Specify all databases, registers, websites, organisations, reference lists and other sources searched or consulted to identify studies. Specify the date when each source was last searched or consulted. | 3 |
| Search strategy | 7 | Present the full search strategies for all databases, registers and websites, including any filters and limits used. | 3 |
| Selection process | 8 | Specify the methods used to decide whether a study met the inclusion criteria of the review, including how many reviewers screened each record and each report retrieved, whether they worked independently, and if applicable, details of automation tools used in the process. | 3 |
| Data collection process | 9 | Specify the methods used to collect data from reports, including how many reviewers collected data from each report, whether they worked independently, any processes for obtaining or confirming data from study investigators, and if applicable, details of automation tools used in the process. | 4 |
| Data items | 10a | List and define all outcomes for which data were sought. Specify whether all results that were compatible with each outcome domain in each study were sought (e.g. for all measures, time points, analyses), and if not, the methods used to decide which results to collect. | 3 |
|  | 10b | List and define all other variables for which data were sought (e.g. participant and intervention characteristics, funding sources). Describe any assumptions made about any missing or unclear information. | 3 |
| Study risk of bias assessment | 11 | Specify the methods used to assess risk of bias in the included studies, including details of the tool(s) used, how many reviewers assessed each study and whether they worked independently, and if applicable, details of automation tools used in the process. | 4 |
| Effect measures | 12 | Specify for each outcome the effect measure(s) (e.g. risk ratio, mean difference) used in the synthesis or presentation of results. | 4 |
| Synthesis methods | 13a | Describe the processes used to decide which studies were eligible for each synthesis (e.g. tabulating the study intervention characteristics and comparing against the planned groups for each synthesis (item #5)). | 4 |
|  | 13b | Describe any methods required to prepare the data for presentation or synthesis, such as handling of missing summary statistics, or data conversions. | 4 |
|  | 13c | Describe any methods used to tabulate or visually display results of individual studies and syntheses. | 4 |
|  | 13d | Describe any methods used to synthesize results and provide a rationale for the choice(s). If meta-analysis was performed, describe the model(s), method(s) to identify the presence and extent of statistical heterogeneity, and software package(s) used. | 4 |
|  | 13e | Describe any methods used to explore possible causes of heterogeneity among study results (e.g. subgroup analysis, meta-regression). | 4 |
|  | 13f | Describe any sensitivity analyses conducted to assess robustness of the synthesized results. | 4 |
| Reporting bias assessment | 14 | Describe any methods used to assess risk of bias due to missing results in a synthesis (arising from reporting biases). | 4 |
| Certainty assessment | 15 | Describe any methods used to assess certainty (or confidence) in the body of evidence for an outcome. | 4 |
| **RESULTS** | | |  |
| Study selection | 16a | Describe the results of the search and selection process, from the number of records identified in the search to the number of studies included in the review, ideally using a flow diagram. | 4-5 |
|  | 16b | Cite studies that might appear to meet the inclusion criteria, but which were excluded, and explain why they were excluded. | 4-5 |
| Study characteristics | 17 | Cite each included study and present its characteristics. | 5 |
| Risk of bias in studies | 18 | Present assessments of risk of bias for each included study. | 6 |
| Results of individual studies | 19 | For all outcomes, present, for each study: (a) summary statistics for each group (where appropriate) and (b) an effect estimate and its precision (e.g. confidence/credible interval), ideally using structured tables or plots. | 6 |
| Results of syntheses | 20a | For each synthesis, briefly summarise the characteristics and risk of bias among contributing studies. | 6 |
|  | 20b | Present results of all statistical syntheses conducted. If meta-analysis was done, present for each the summary estimate and its precision (e.g. confidence/credible interval) and measures of statistical heterogeneity. If comparing groups, describe the direction of the effect. | 6-7 |
|  | 20c | Present results of all investigations of possible causes of heterogeneity among study results. | 6-7 |
|  | 20d | Present results of all sensitivity analyses conducted to assess the robustness of the synthesized results. | 6-7 |
| Reporting biases | 21 | Present assessments of risk of bias due to missing results (arising from reporting biases) for each synthesis assessed. | 7 |
| Certainty of evidence | 22 | Present assessments of certainty (or confidence) in the body of evidence for each outcome assessed. | 7 |
| **DISCUSSION** | | |  |
| Discussion | 23a | Provide a general interpretation of the results in the context of other evidence. | 7-9 |
|  | 23b | Discuss any limitations of the evidence included in the review. | 9-10 |
|  | 23c | Discuss any limitations of the review processes used. | 9-10 |
|  | 23d | Discuss implications of the results for practice, policy, and future research. | 10-11 |
| **OTHER INFORMATION** | | |  |
| Registration and protocol | 24a | Provide registration information for the review, including register name and registration number, or state that the review was not registered. | 3 |
|  | 24b | Indicate where the review protocol can be accessed, or state that a protocol was not prepared. | 3 |
|  | 24c | Describe and explain any amendments to information provided at registration or in the protocol. | 3 |
| Support | 25 | Describe sources of financial or non-financial support for the review, and the role of the funders or sponsors in the review. | 11 |
| Competing interests | 26 | Declare any competing interests of review authors. | 11 |
| Availability of data, code and other materials | 27 | Report which of the following are publicly available and where they can be found: template data collection forms; data extracted from included studies; data used for all analyses; analytic code; any other materials used in the review. | 11 |

*From:*  Page MJ, McKenzie JE, Bossuyt PM, Boutron I, Hoffmann TC, Mulrow CD, et al. The PRISMA 2020 statement: an updated guideline for reporting systematic reviews. BMJ 2021;372:n71. doi: 10.1136/bmj.n71. This work is licensed under CC BY 4.0. To view a copy of this license, visit <https://creativecommons.org/licenses/by/4.0/>

Table S4. Meta-Regression Analyses for Dose-Response Relationships on Glycemic Outcomes.

| **Outcome** | **Moderator Variable** | **k** | **Coefficient (Slope)** | **95% Confidence Interval** | **p-value** |
| --- | --- | --- | --- | --- | --- |
| HbA1c (%) | Intervention duration (weeks) | 6 | -0.021 | [-0.101, 0.059] | 0.613 |
|  | Sprint time per session (s) | 6 | -0.009 | [-0.080, 0.062] | 0.799 |
|  | Sprint time per week (s) | 6 | -0.003 | [-0.026, 0.020] | 0.799 |
|  | MET-minutes per week | 6 | 0.002 | [-0.010, 0.014] | 0.759 |
| HOMA-IR | Intervention duration (weeks) | 8 | -0.085 | [-0.222, 0.052] | 0.224 |
|  | Sprint time per session (s) | 8 | -0.002 | [-0.008, 0.004] | 0.402 |
|  | Sprint time per week (s) | 8 | -0.001 | [-0.003, 0.001] | 0.402 |
|  | MET-minutes per week | 8 | 0 | [-0.008, 0.008] | 0.976 |
| Fasting Insulin (mU/L) | Intervention duration (weeks) | 8 | -0.032 | [-0.693, 0.629] | 0.924 |
|  | Sprint time per session (s) | 8 | 0.002 | [-0.027, 0.031] | 0.906 |
|  | Sprint time per week (s) | 8 | 0.001 | [-0.009, 0.011] | 0.906 |
|  | MET-minutes per week | 8 | 0.004 | [-0.027, 0.035] | 0.783 |
| Fasting Glucose (mmol/L) | Intervention duration (weeks) | 9 | -0.032 | [-0.146, 0.082] | 0.577 |
|  | Sprint time per session (s) | 9 | 0 | [-0.004, 0.004] | 0.774 |
|  | Sprint time per week (s) | 9 | 0 | [-0.002, 0.002] | 0.774 |
|  | MET-minutes per week | 9 | 0 | [-0.006, 0.006] | 0.864 |

k = number of studies included in the meta-regression analysis. Coefficient (Slope) = The estimated change in the pooled Mean Difference (MD) for every one-unit increase in the moderator variable. 95% CI = 95% Confidence Interval (calculated as Coefficient ± 1.96 * Std.Error). All p-values were > 0.20, indicating no statistically significant dose-response relationships were detected within the range of doses included in the analyzed studies.


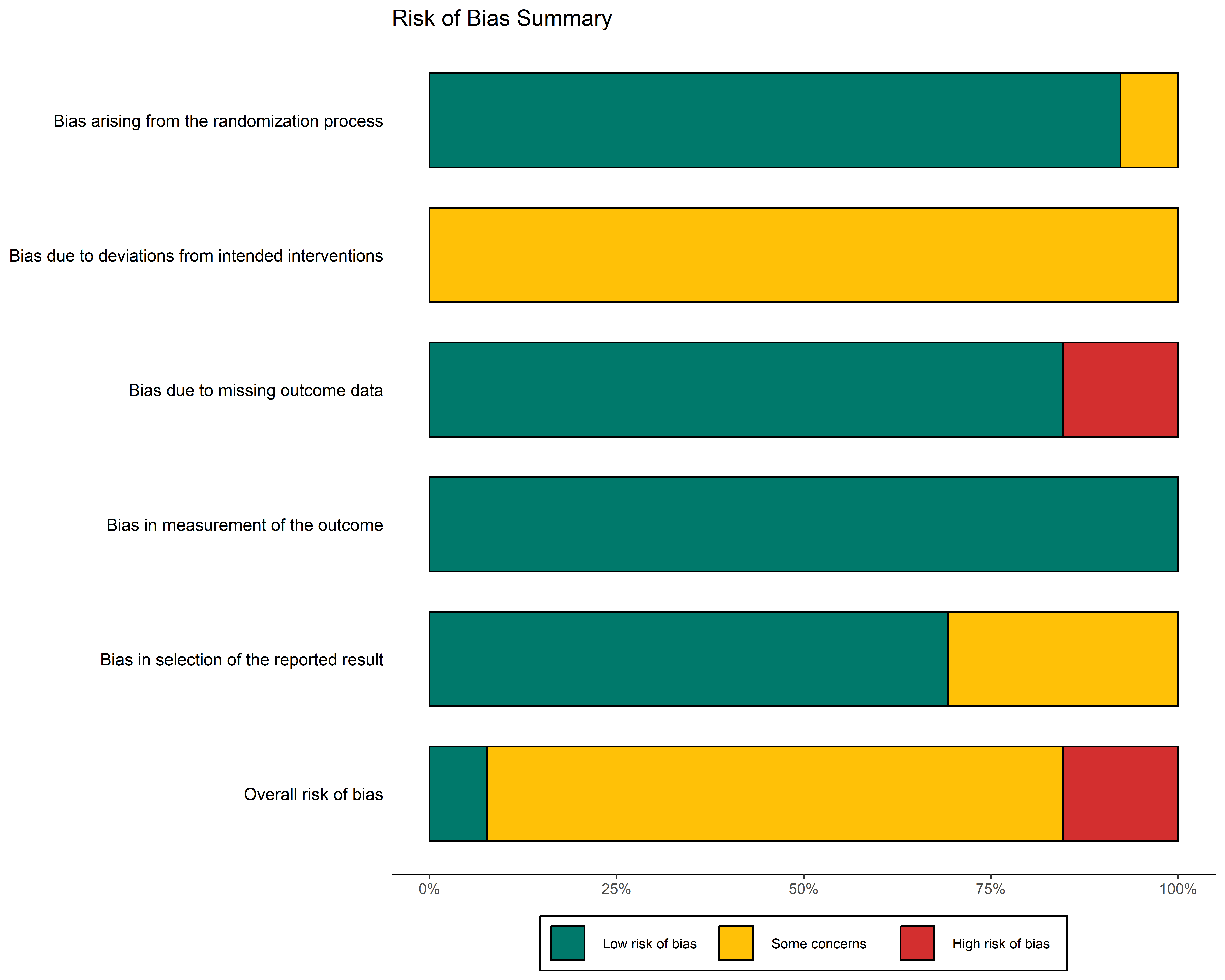


# Figure S1. Risk of bias summary. The bar chart displays the distribution of risk of bias judgments (low risk, some concerns, high risk) for each domain of the Cochrane RoB 2 tool across all included studies.


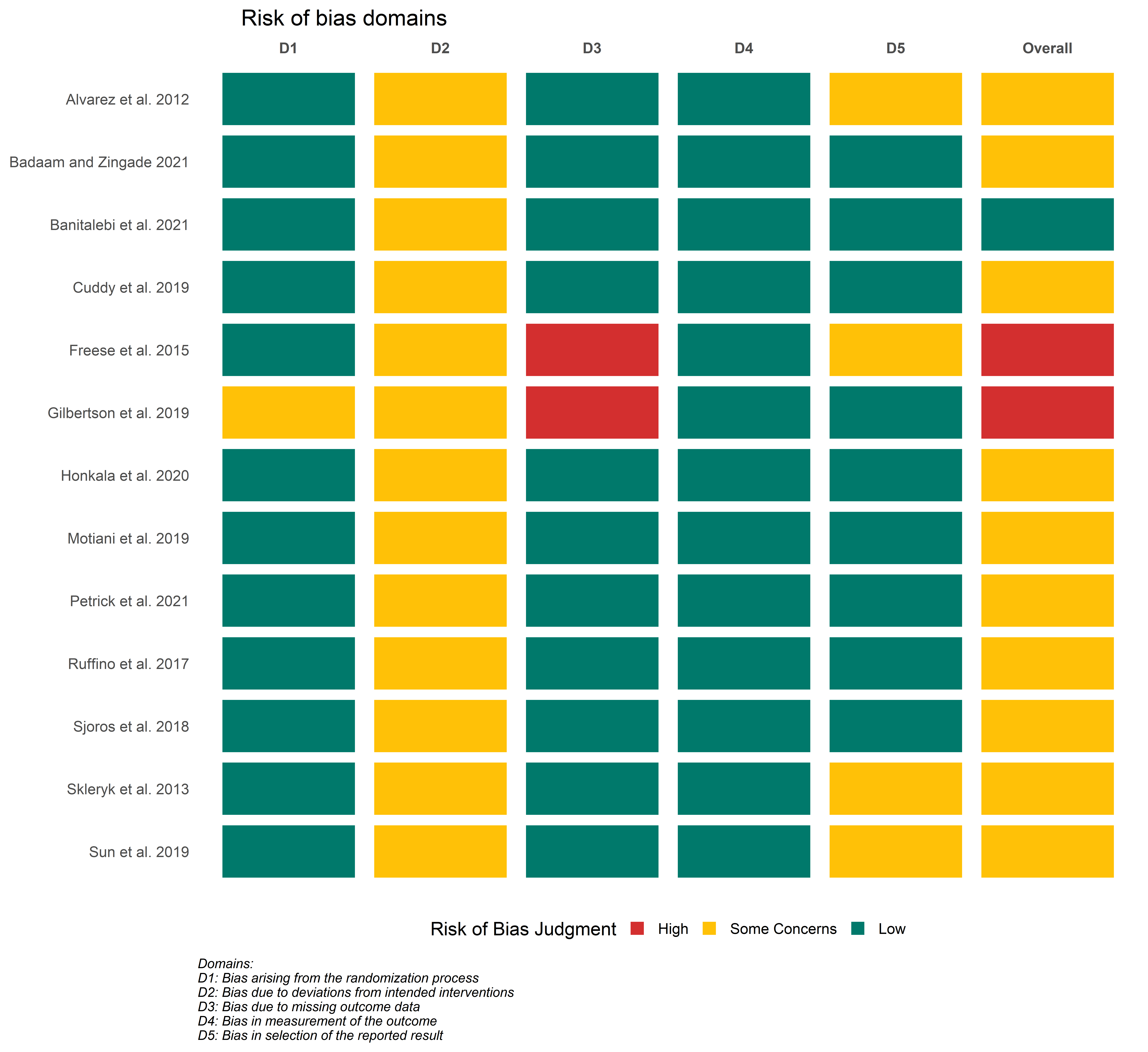


# Figure S2. Risk of bias traffic light plot. Each row represents an individual study, and each column represents a specific risk of bias domain from the Cochrane RoB 2 tool. Green, yellow, and red indicate low risk, some concerns, and high risk of bias, respectively.


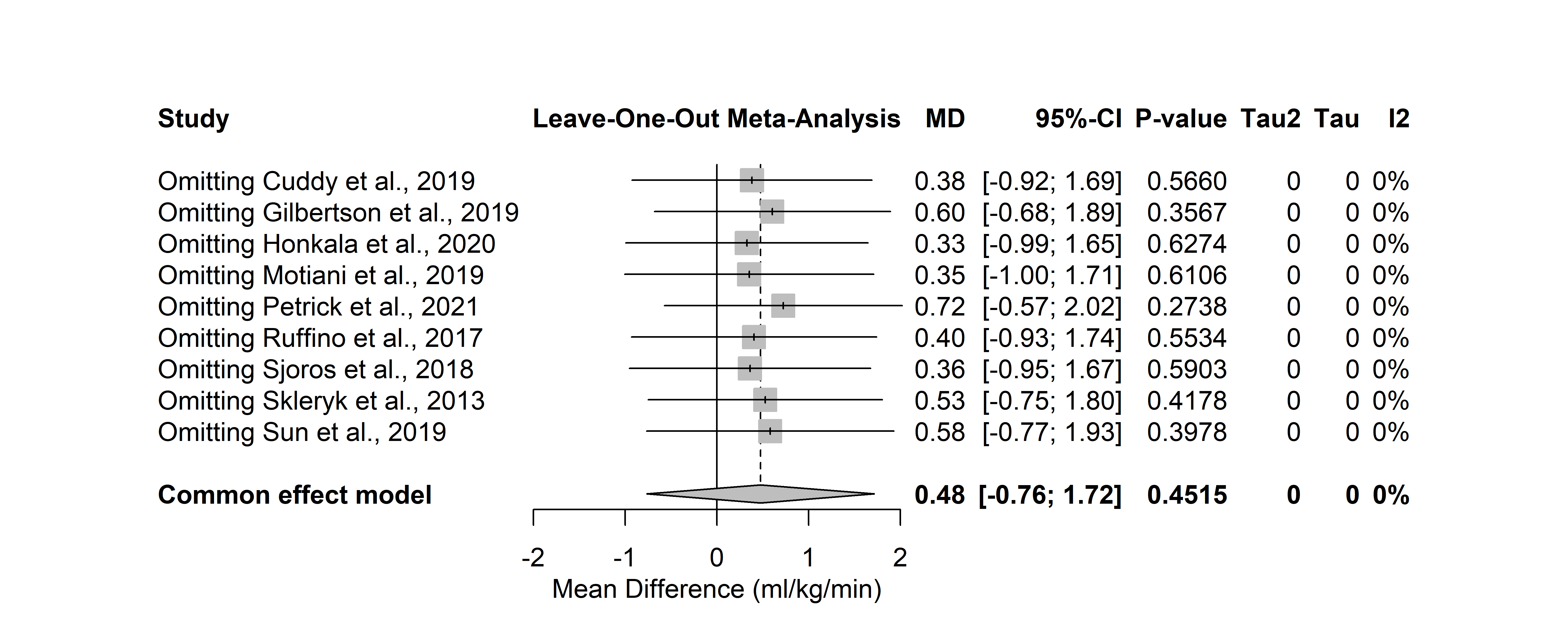

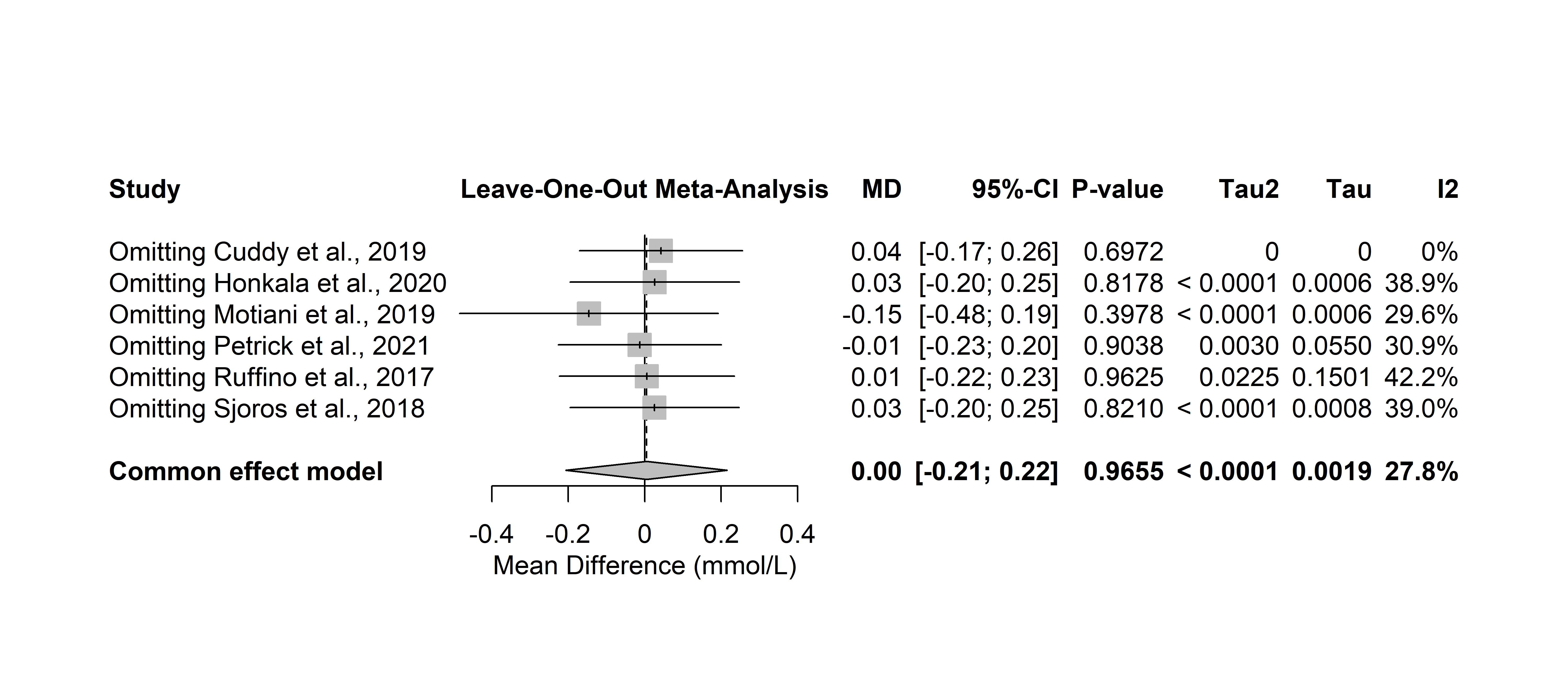

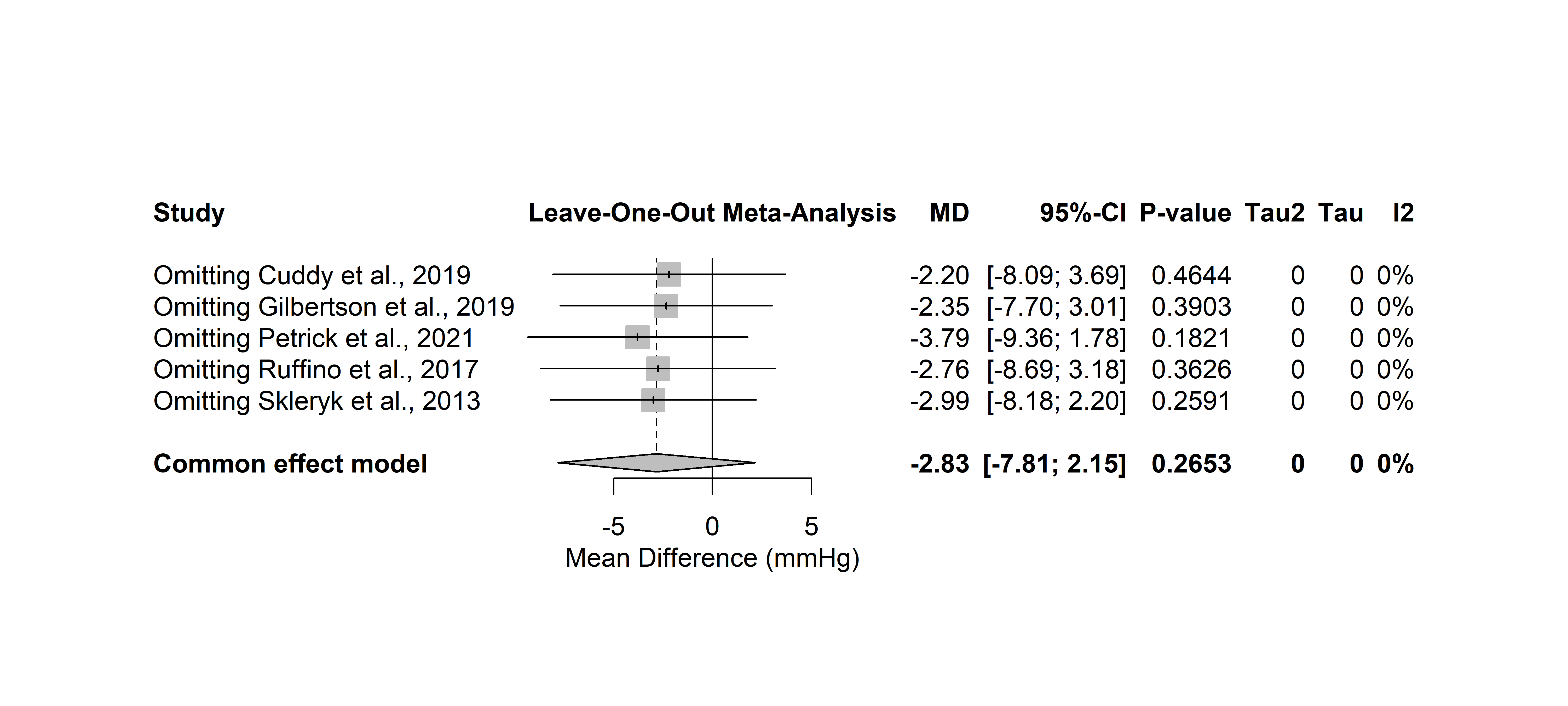

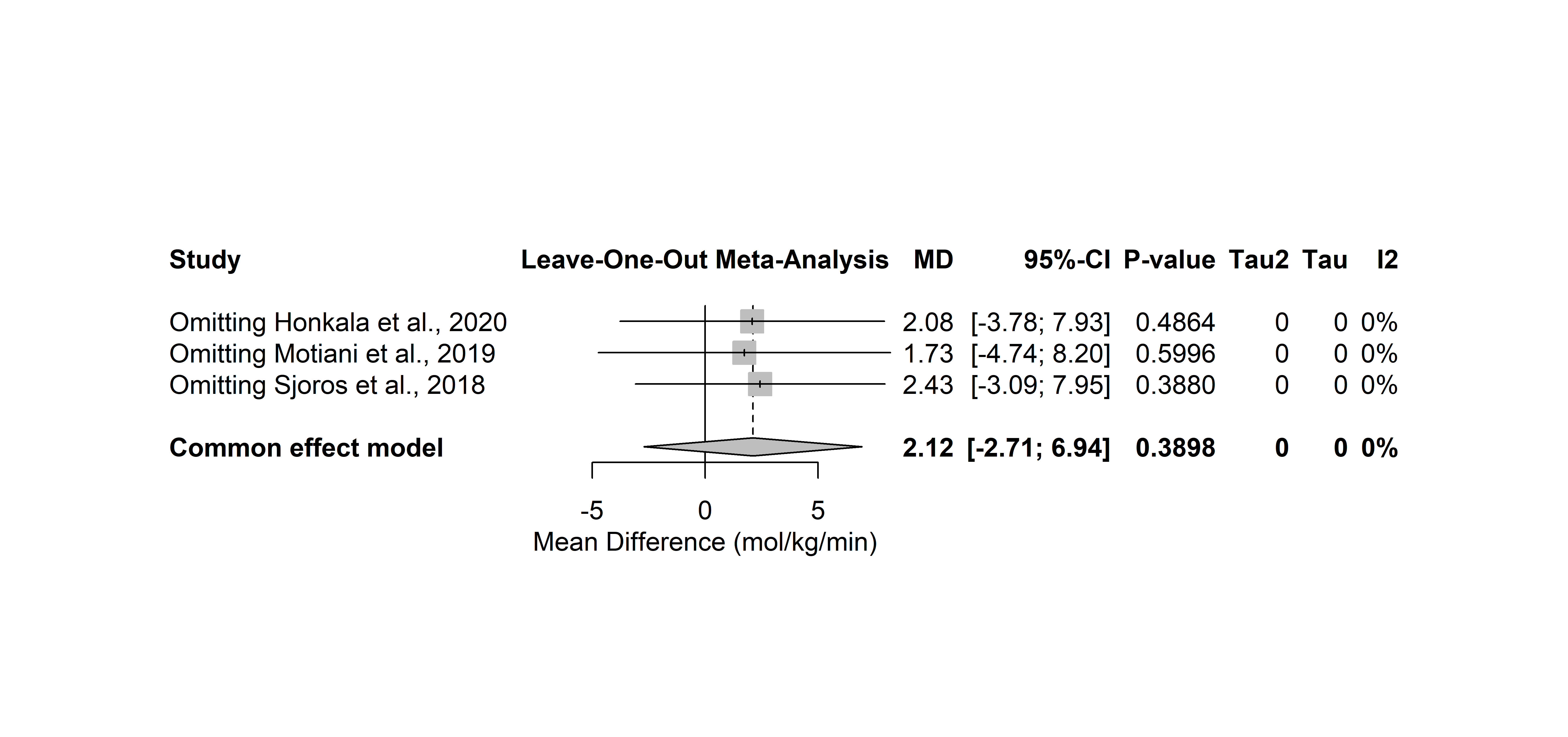

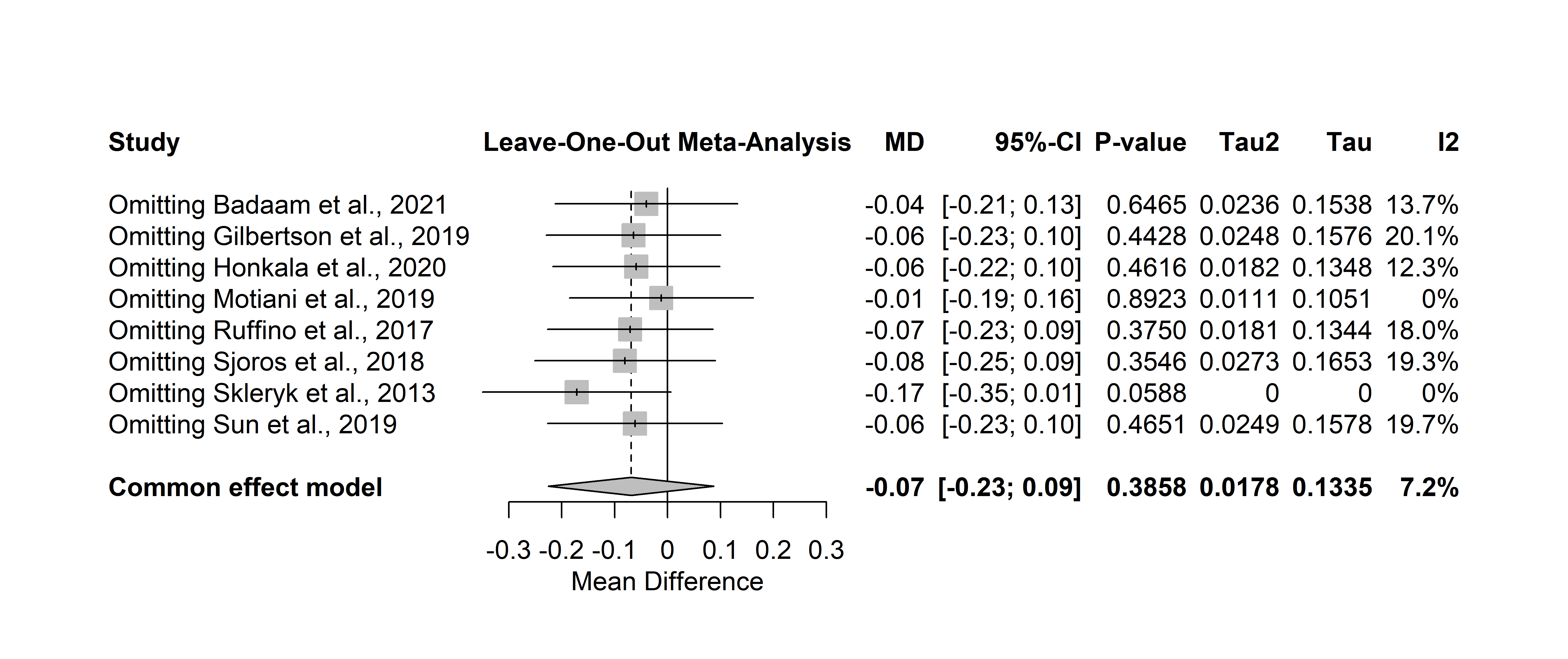

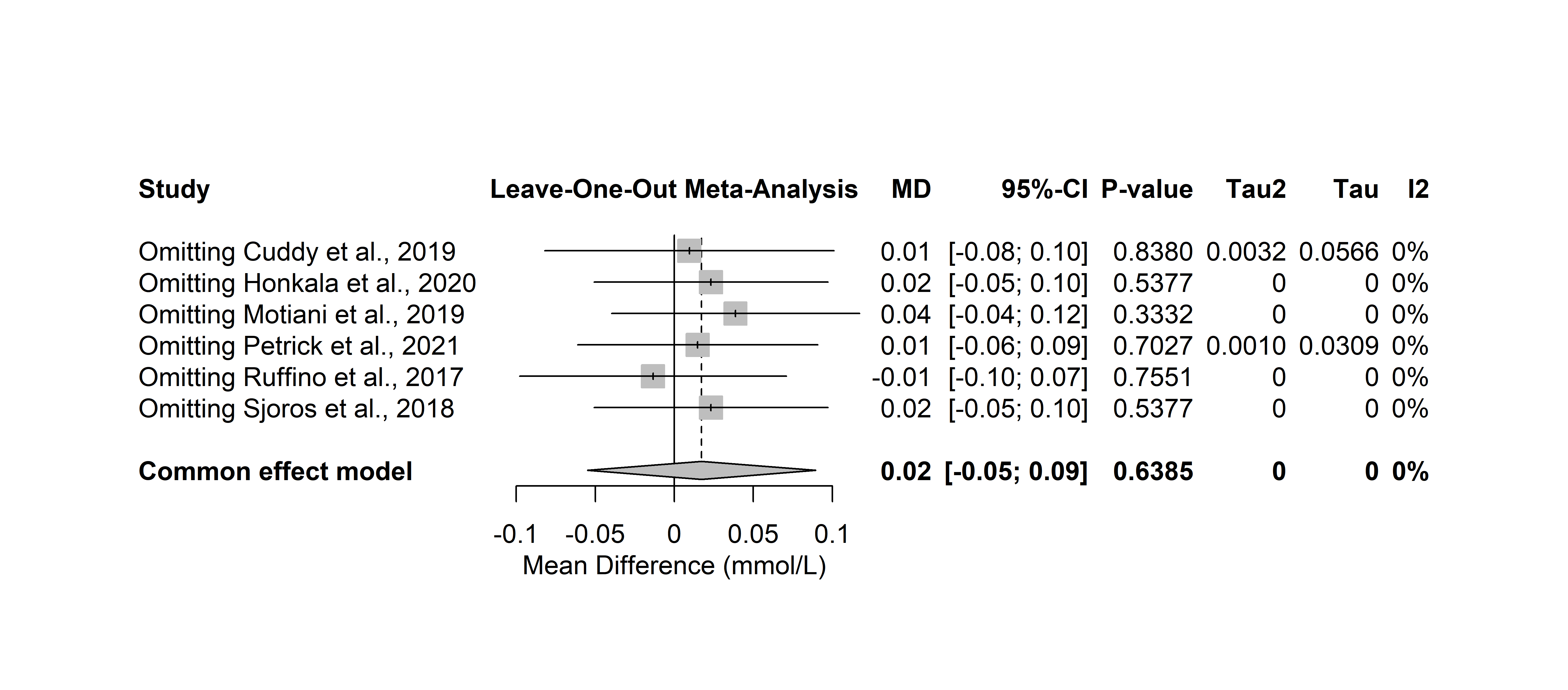

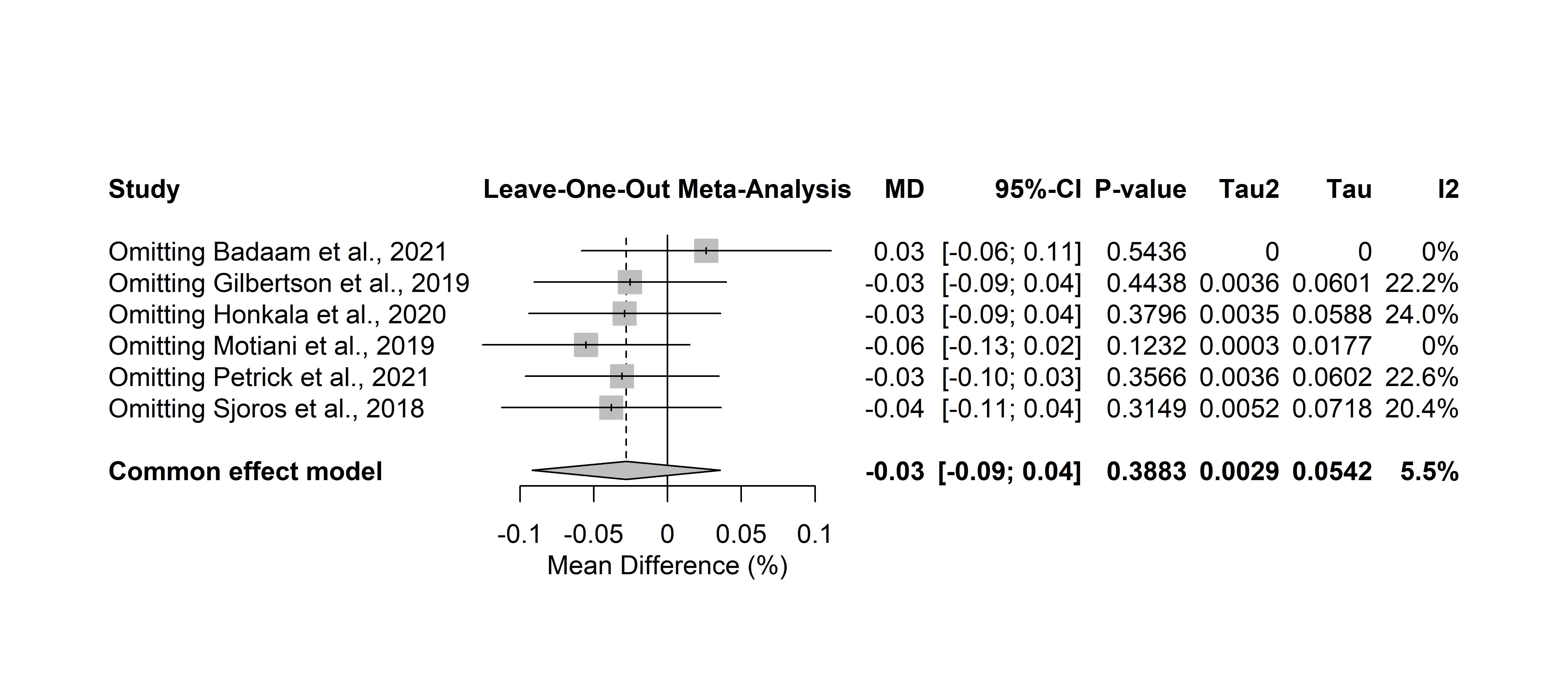

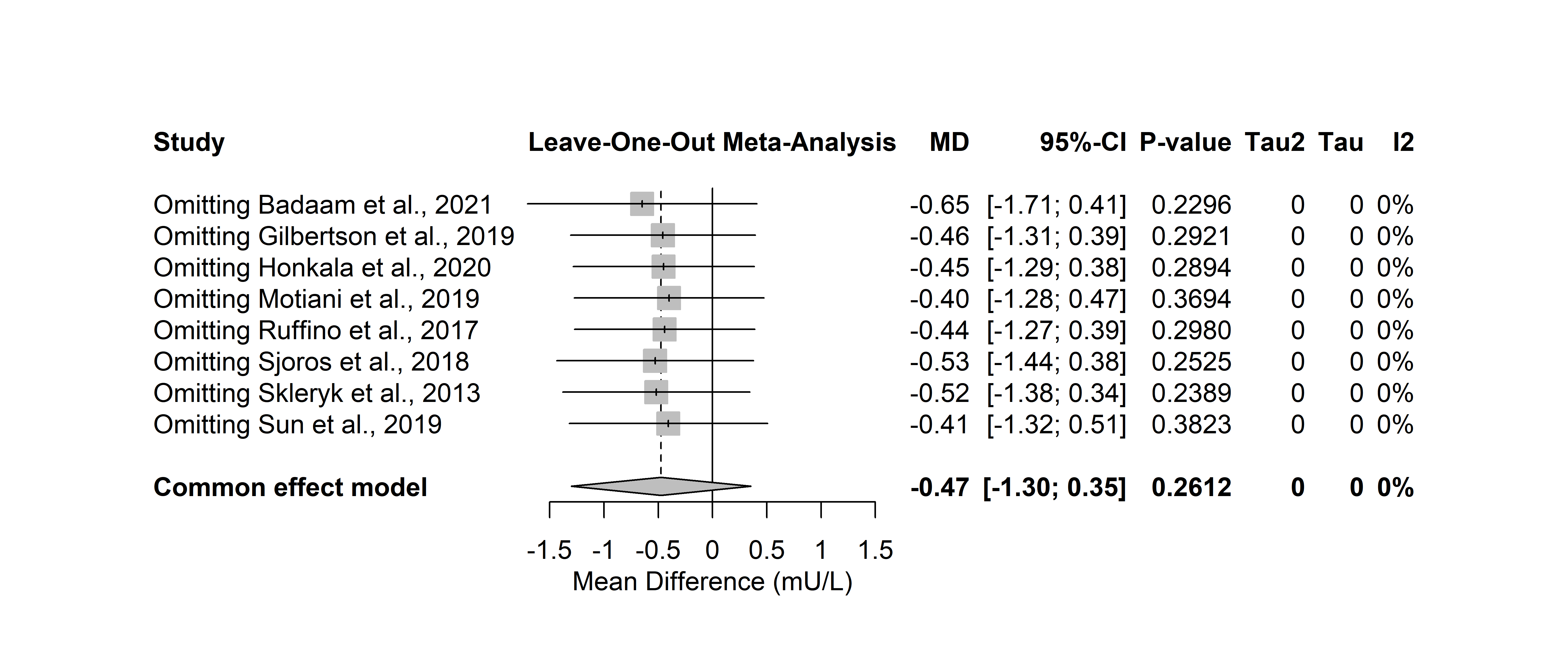

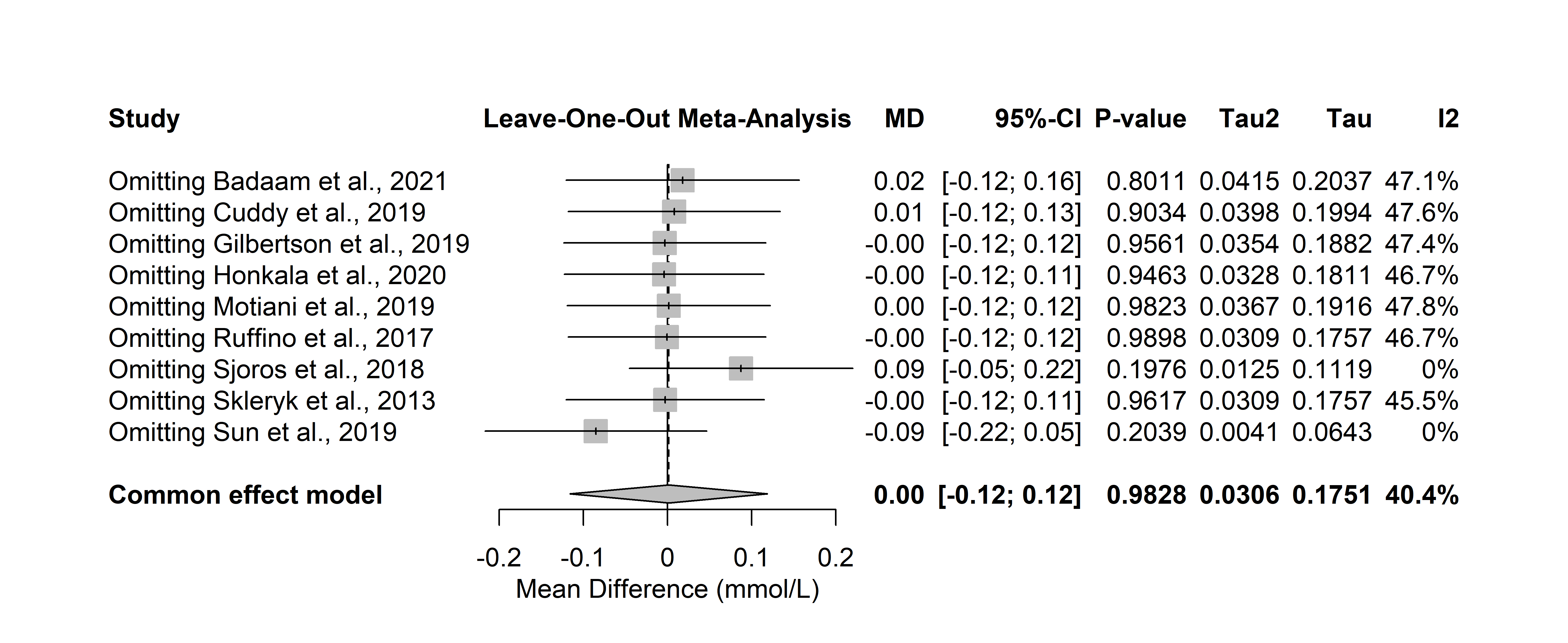

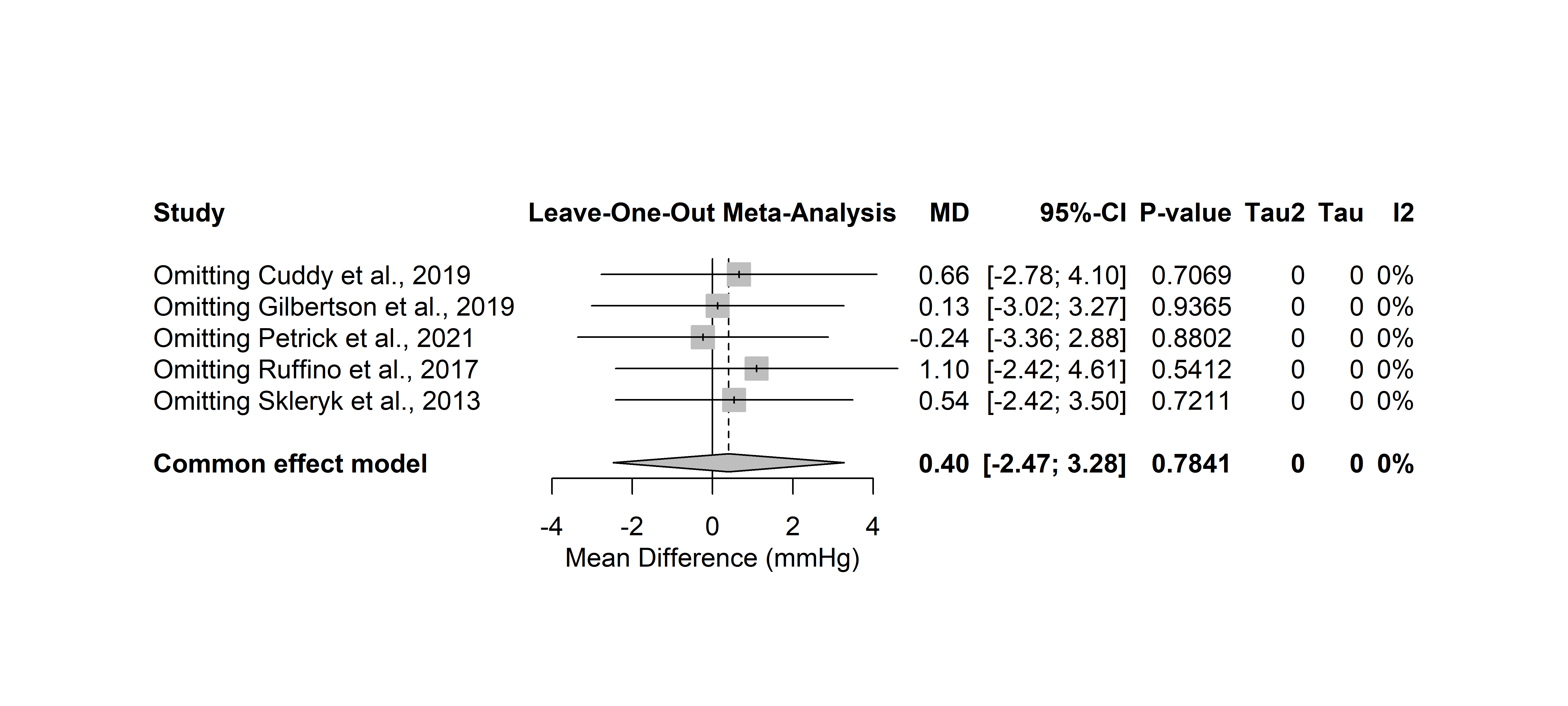

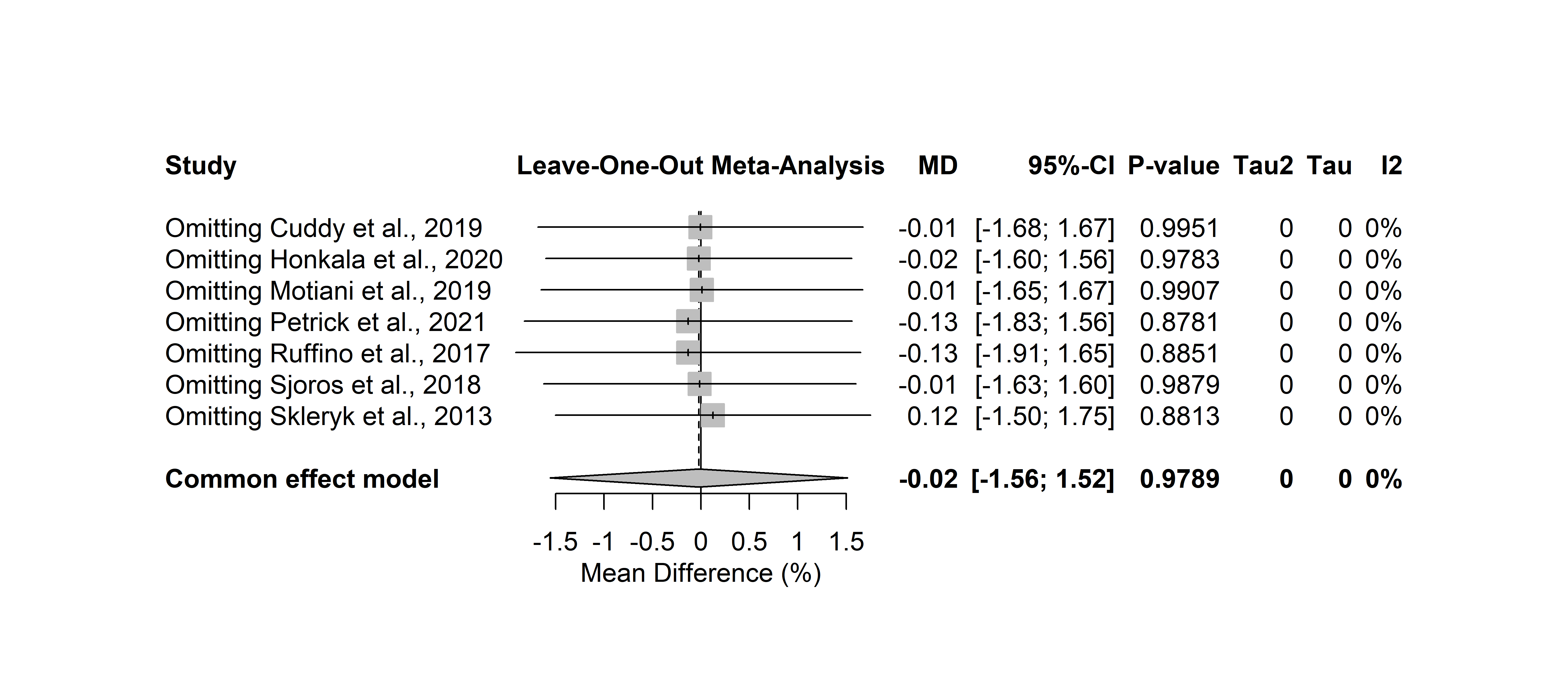

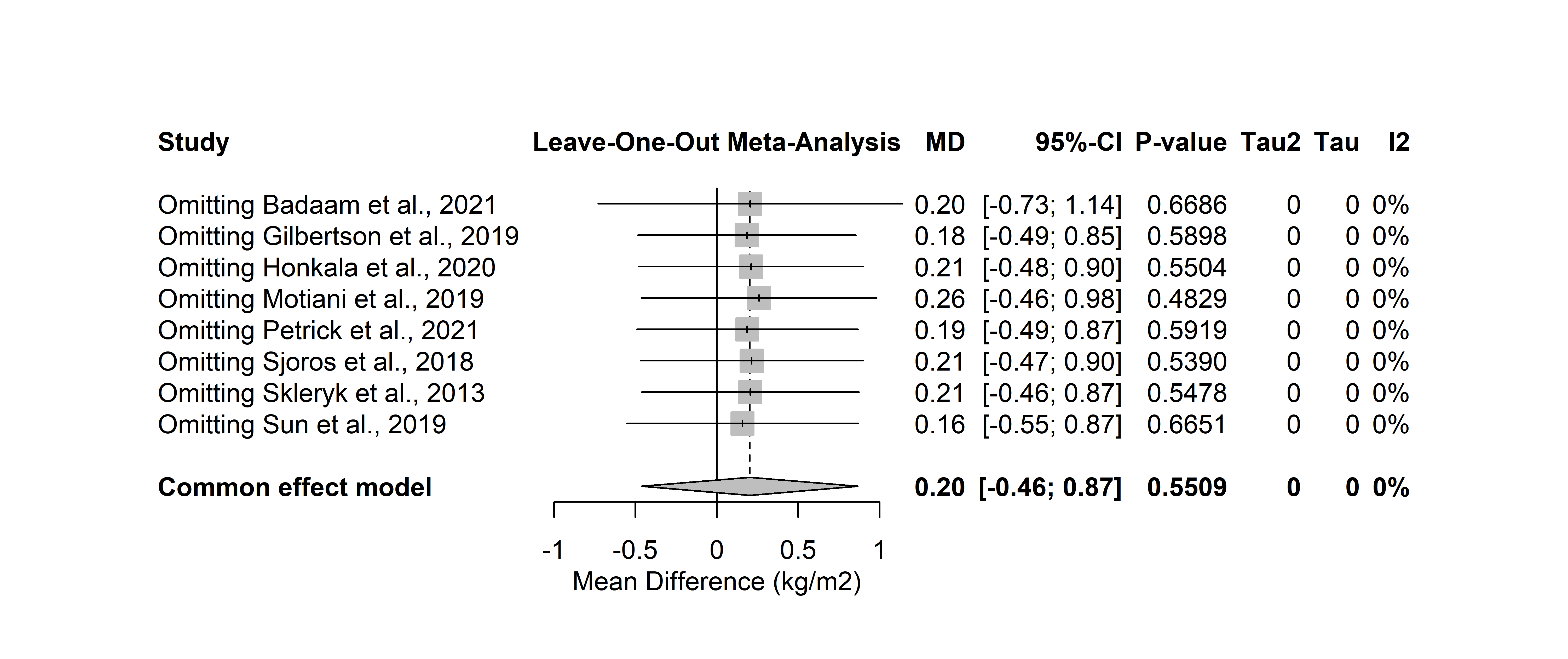

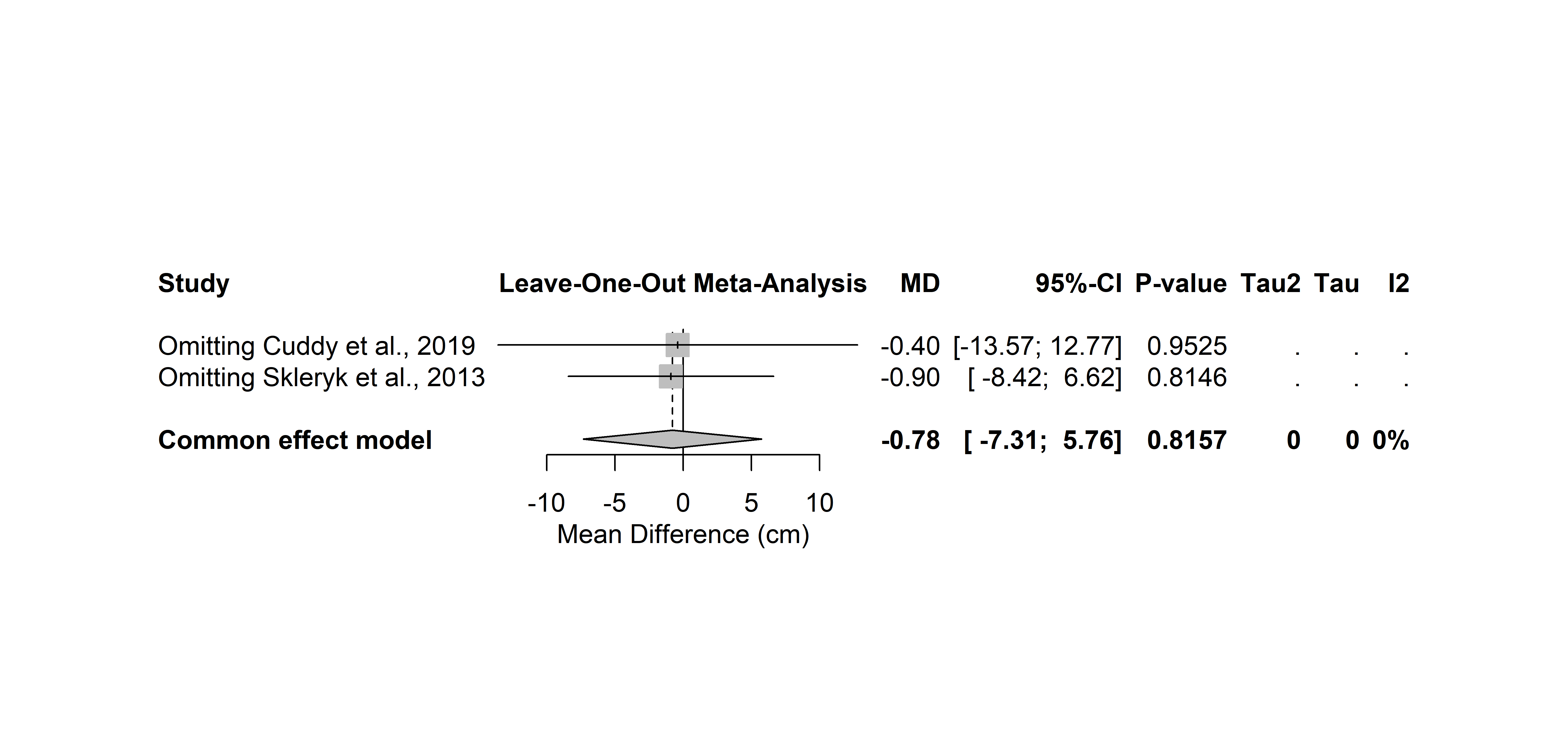


# **Figures S3-S18. Leave-one-out sensitivity analyses.** Each plot corresponds to a specific outcome. Each point and horizontal line represents the pooled mean difference and 95% confidence interval after omitting the study listed on the y-axis. This analysis assesses the influence of individual studies on the overall pooled estimate.


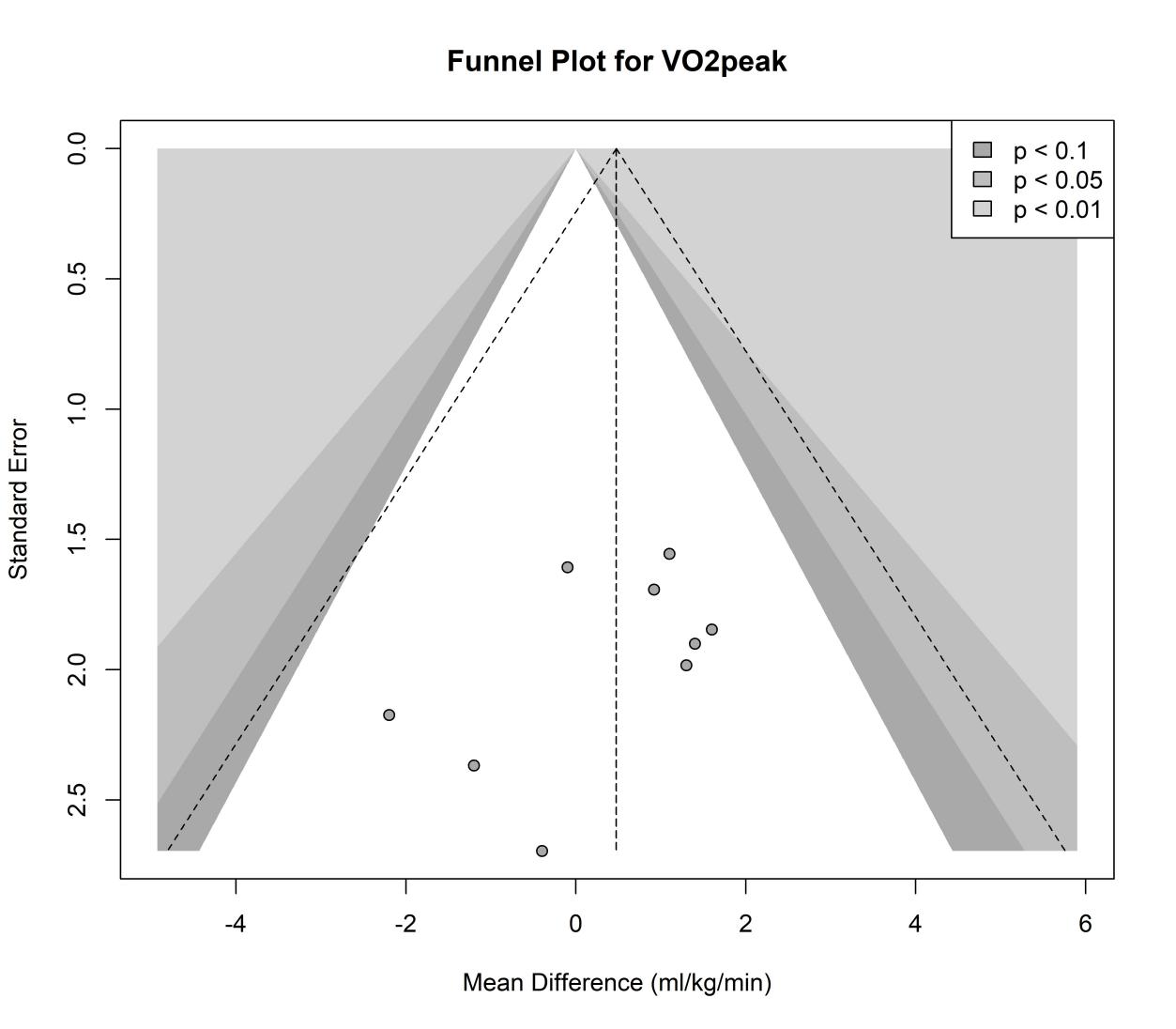

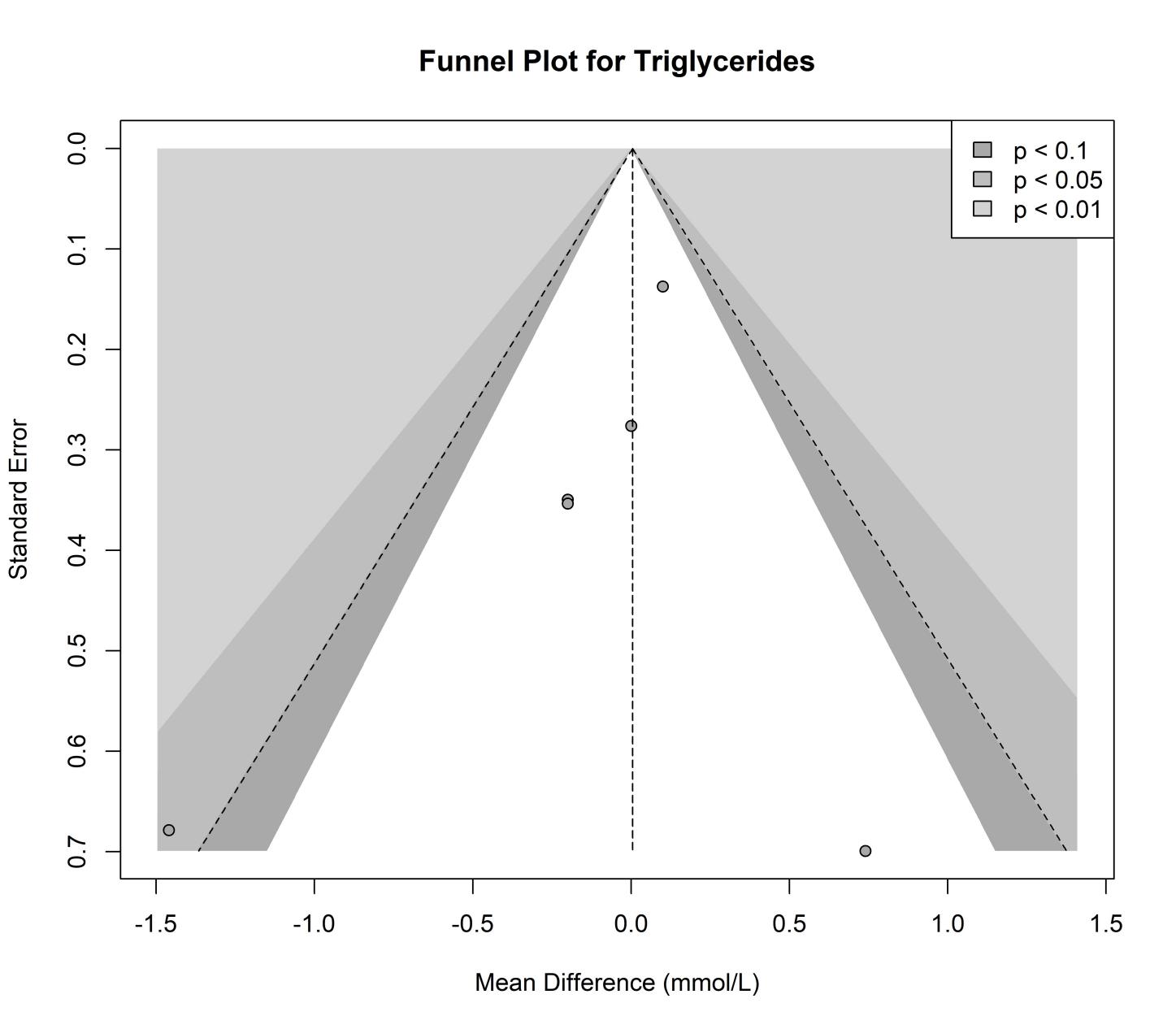

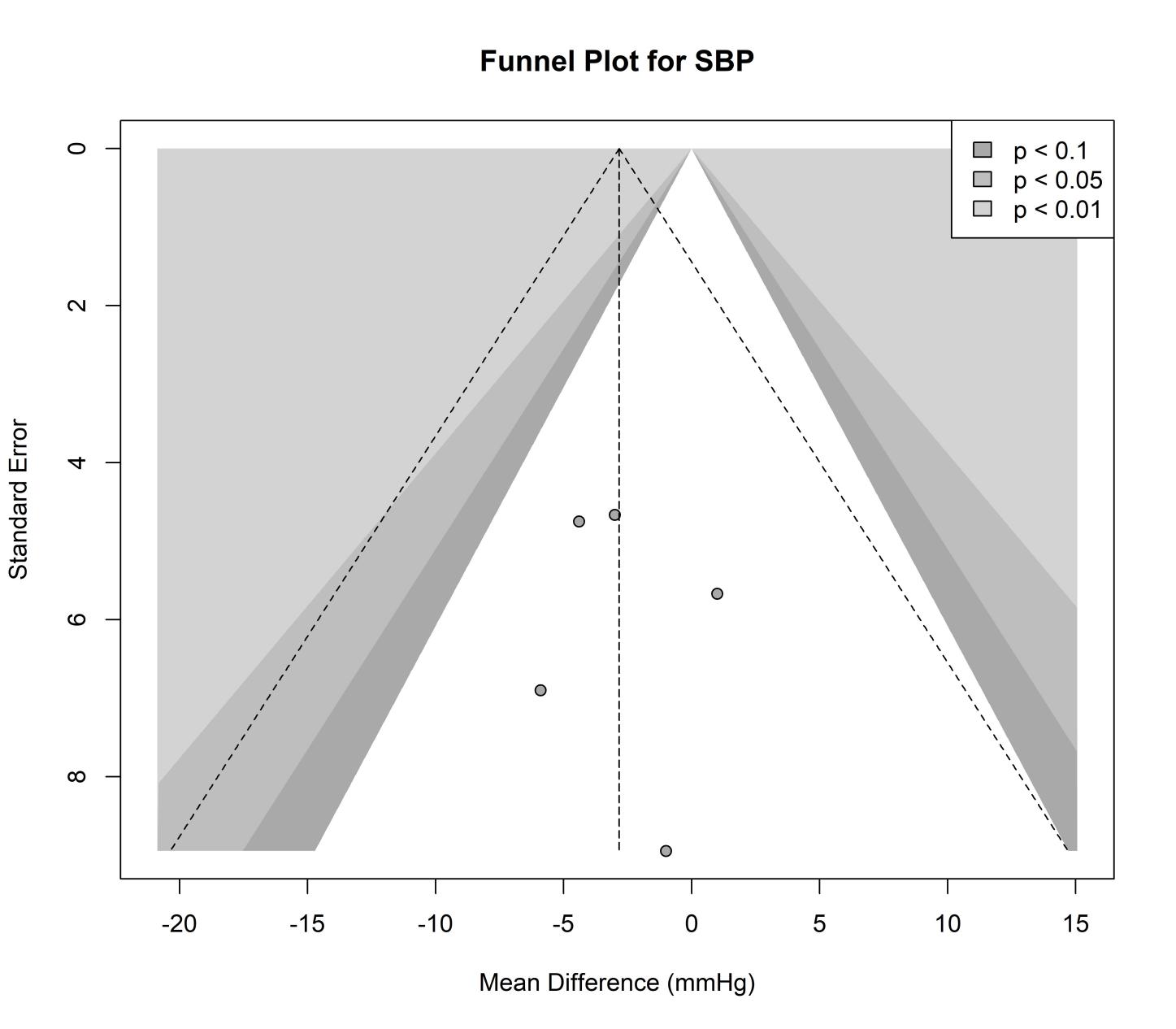

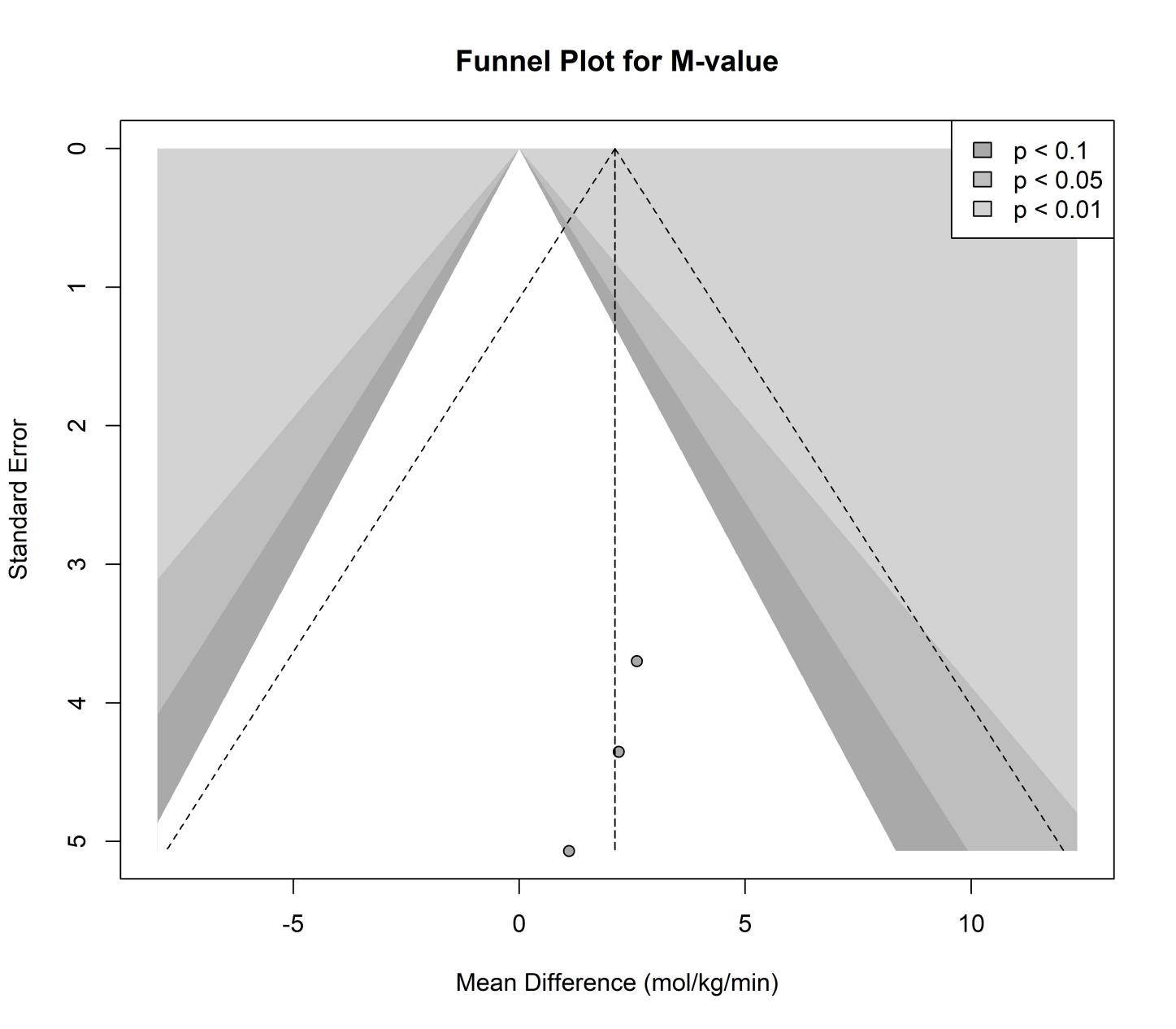

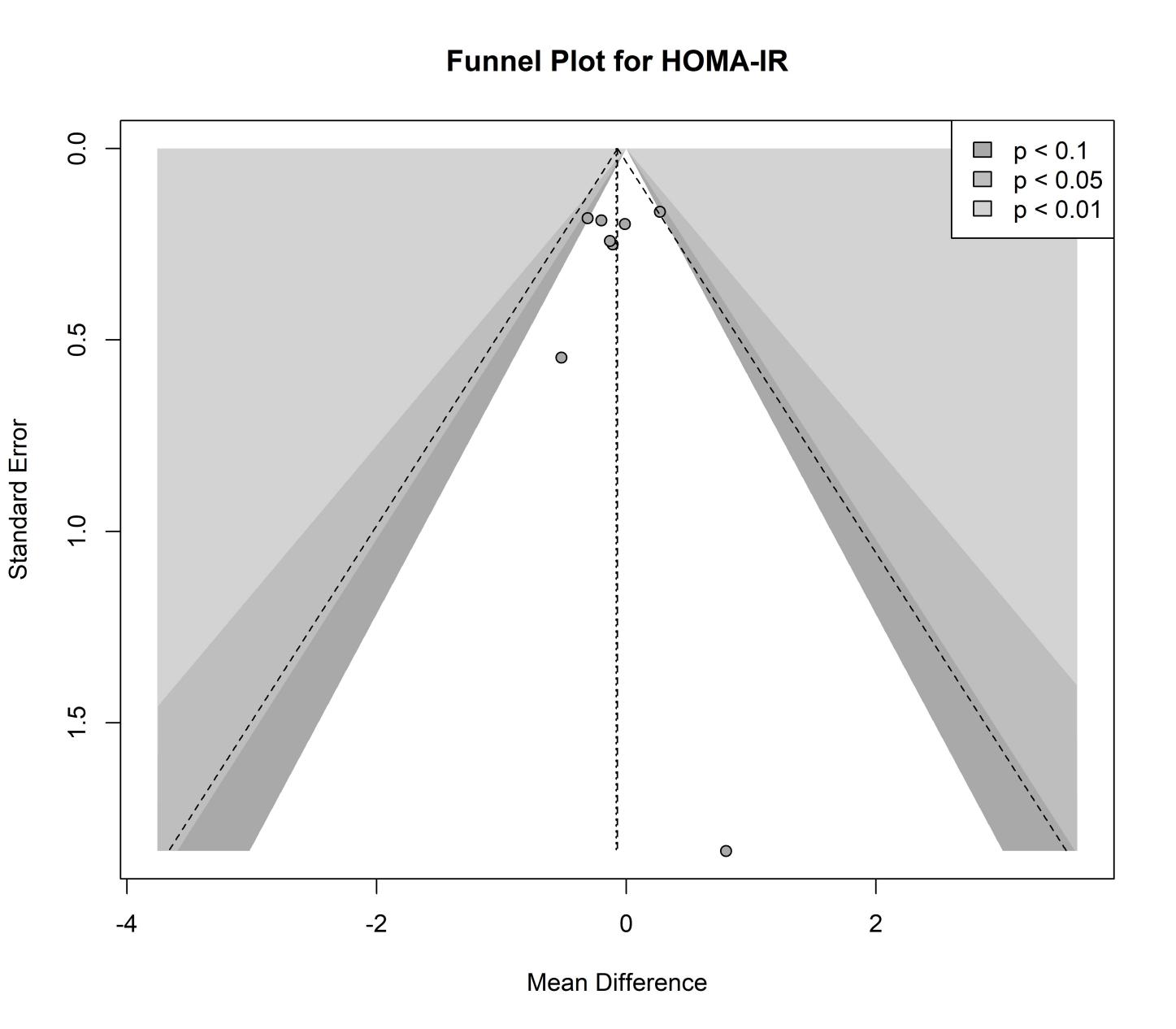

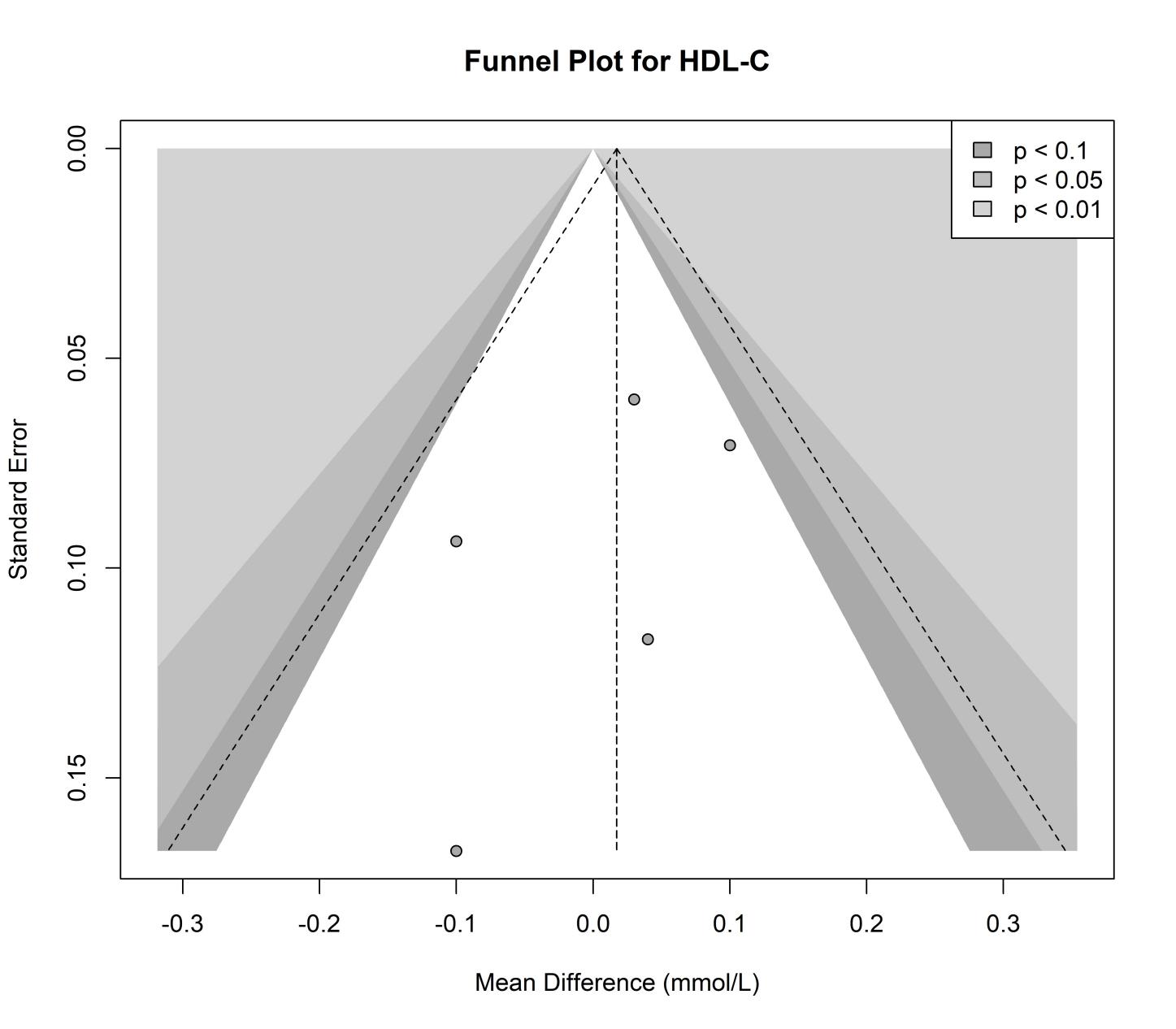

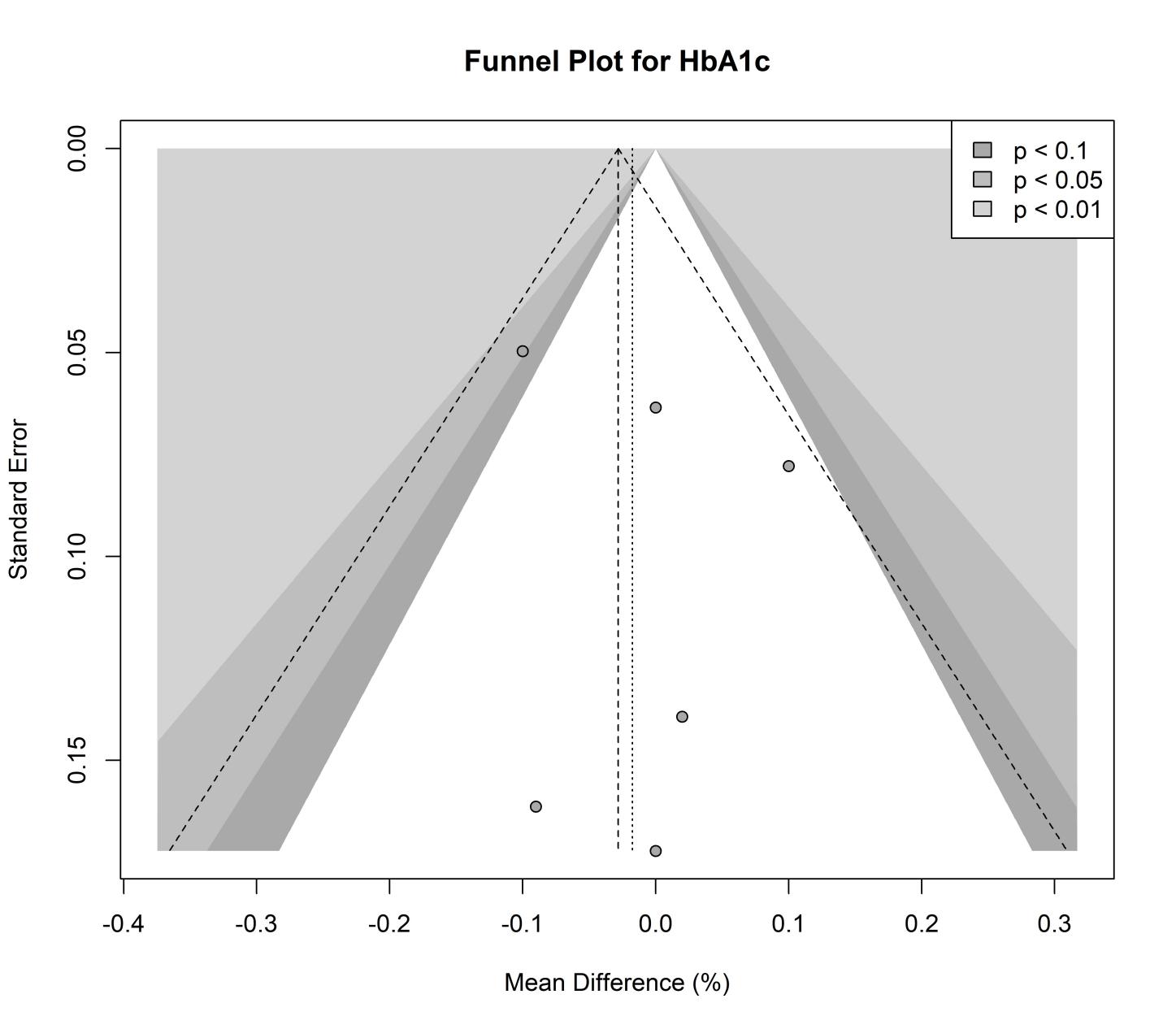

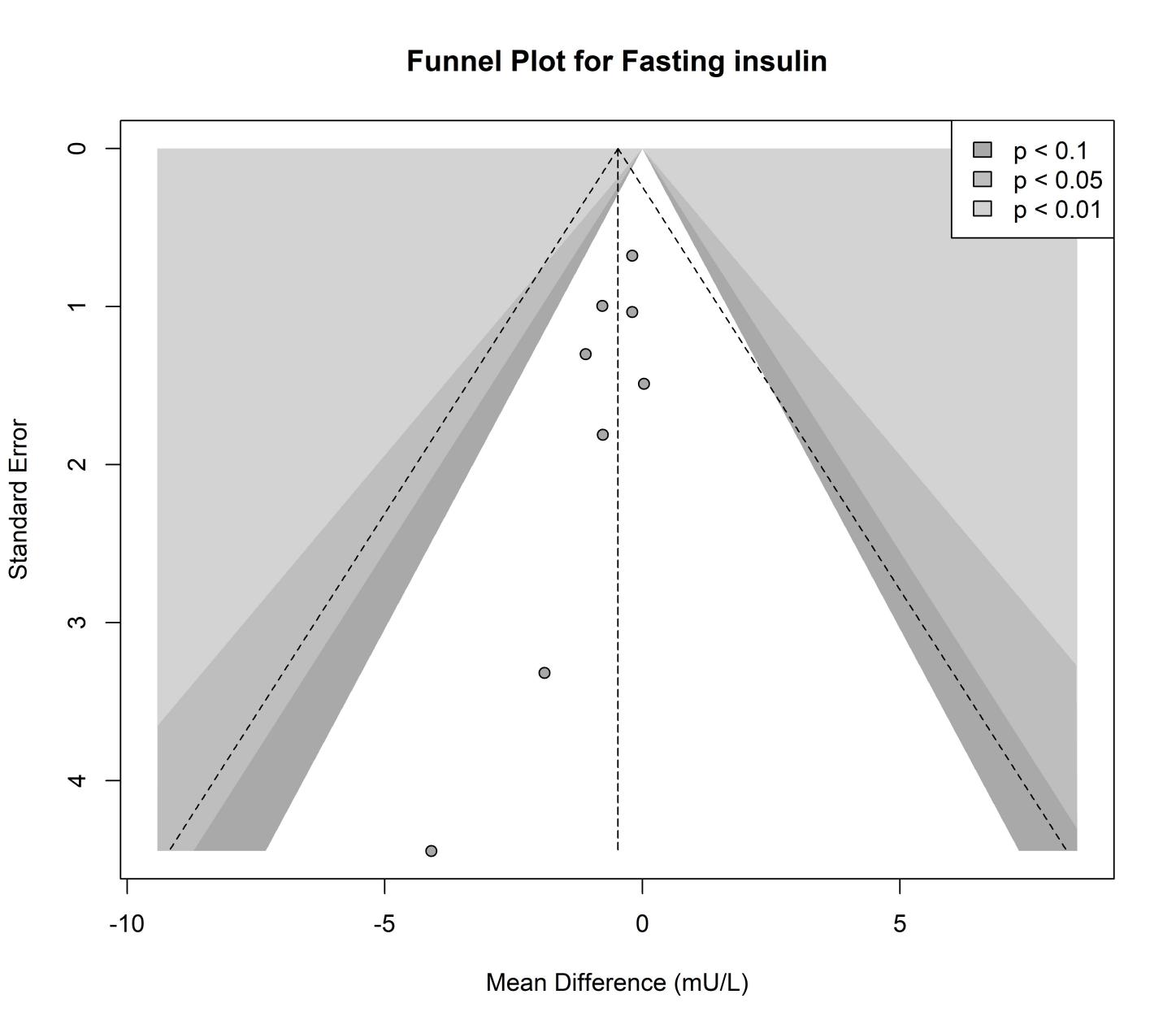

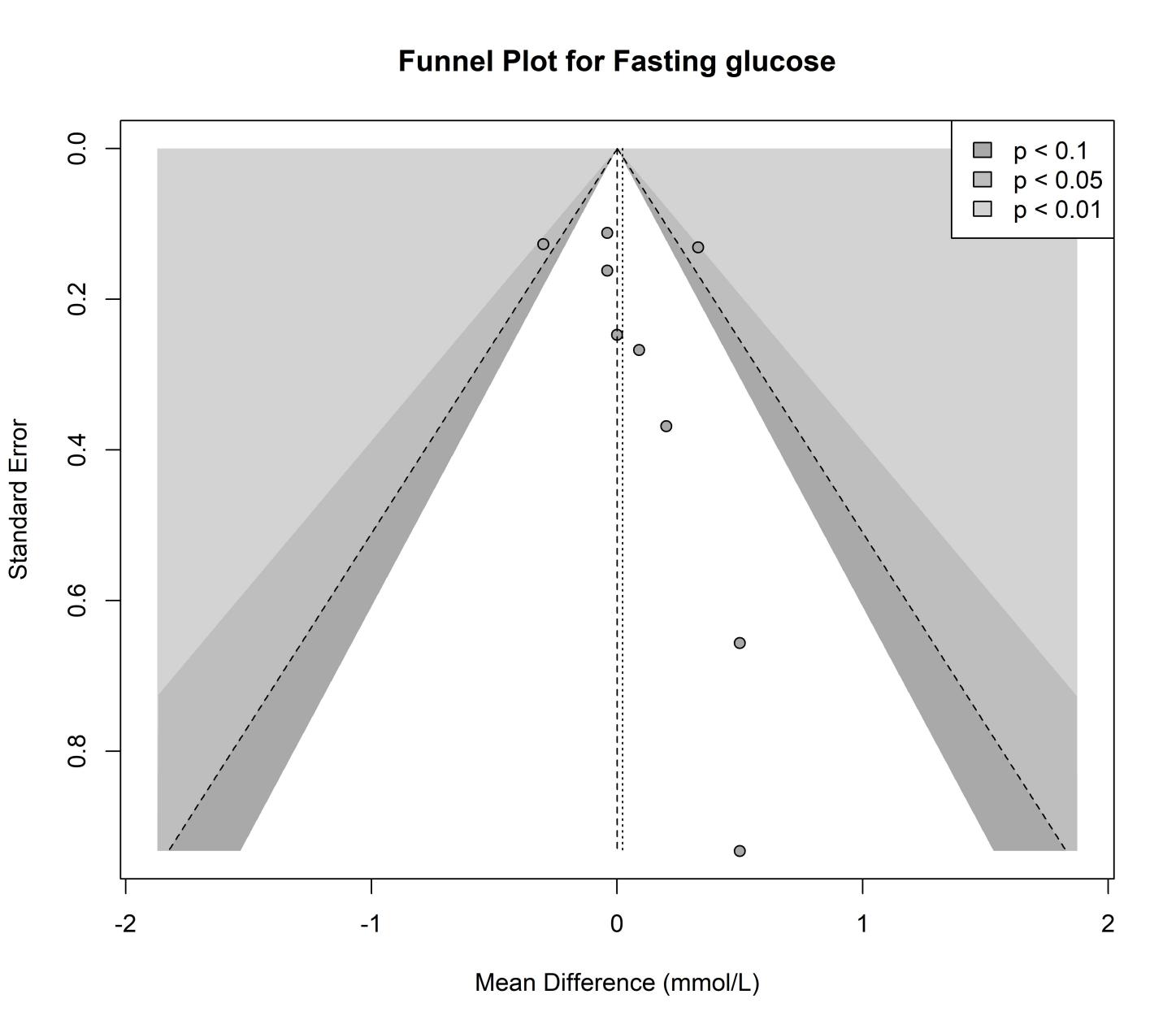

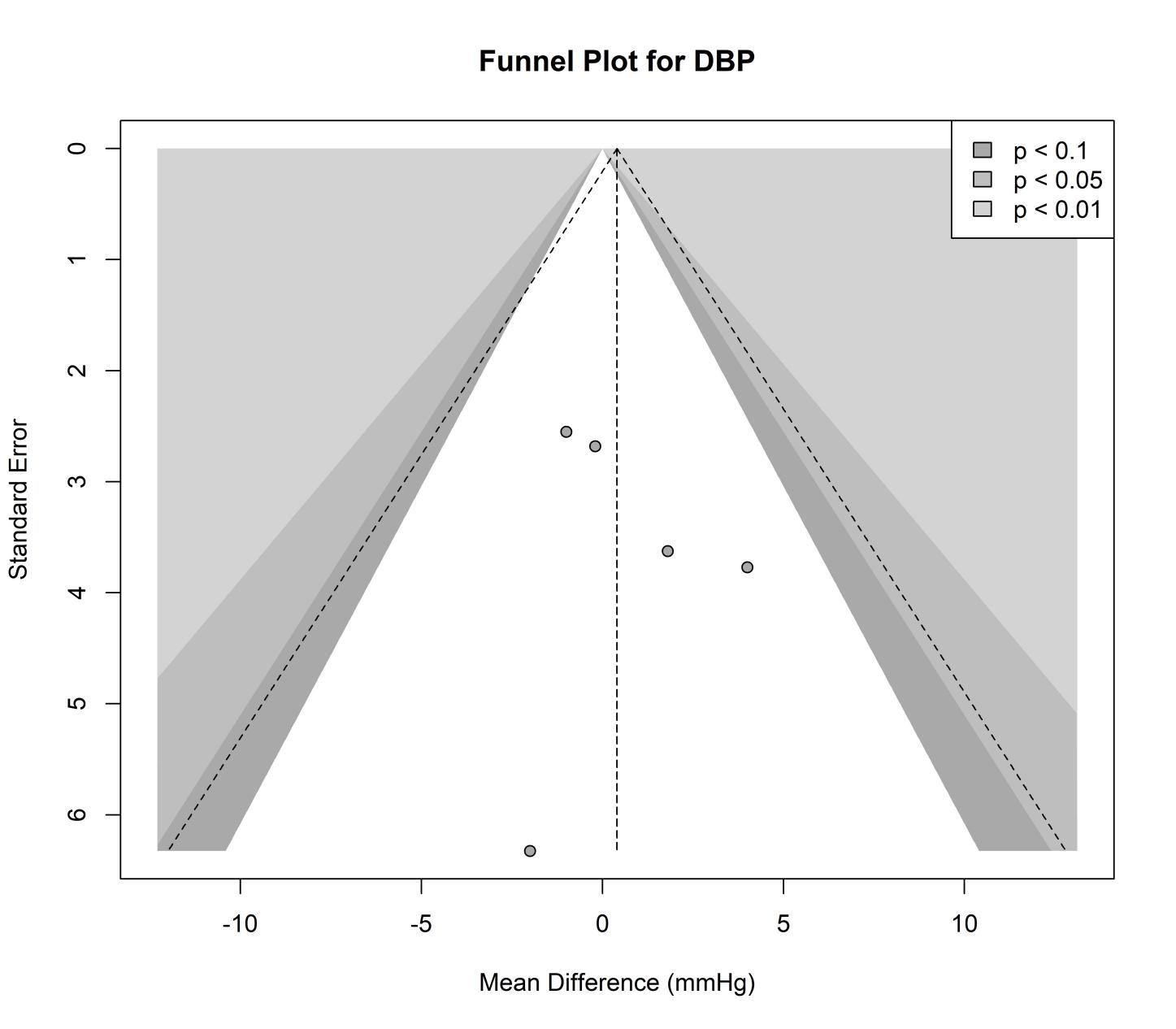

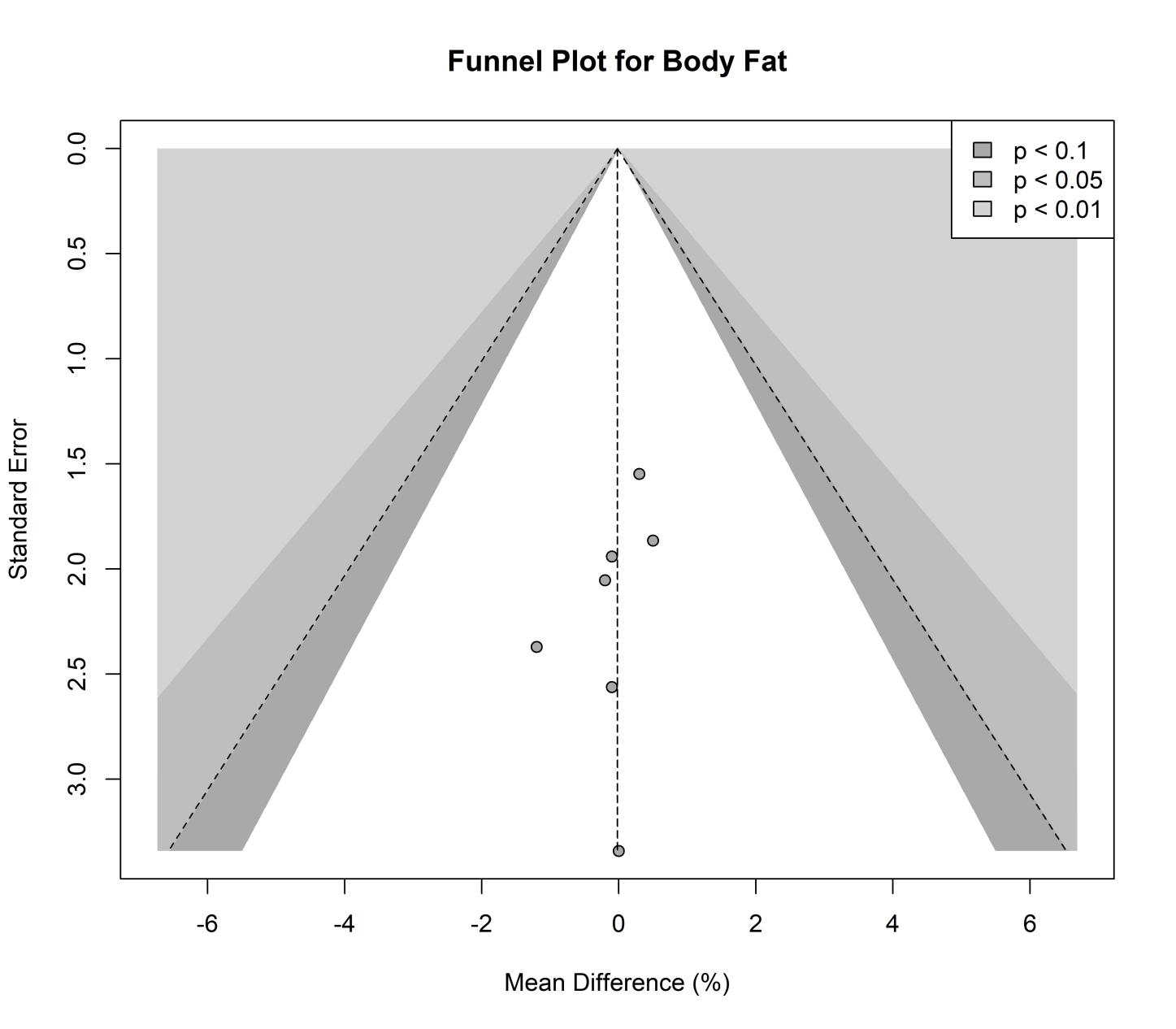

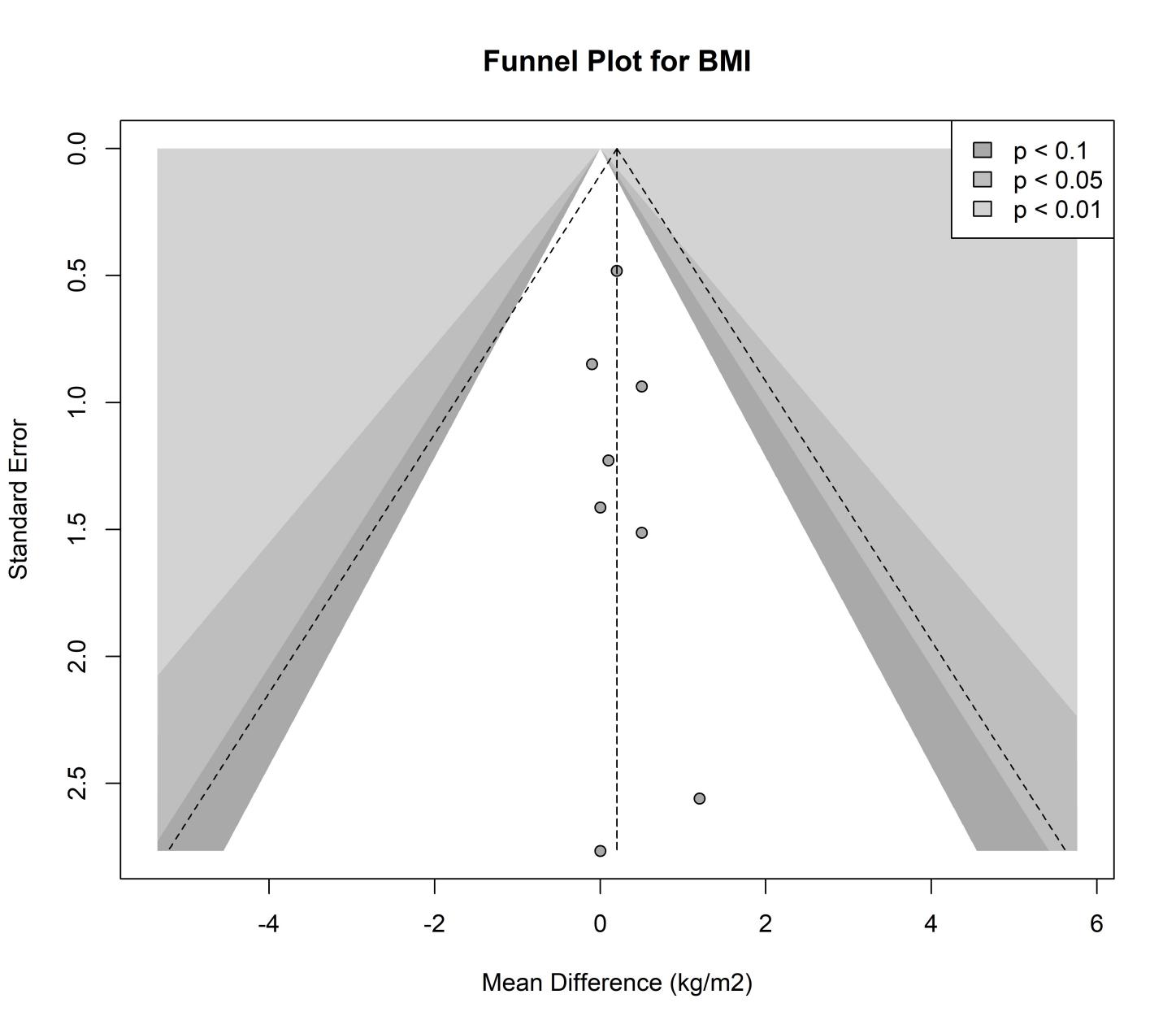

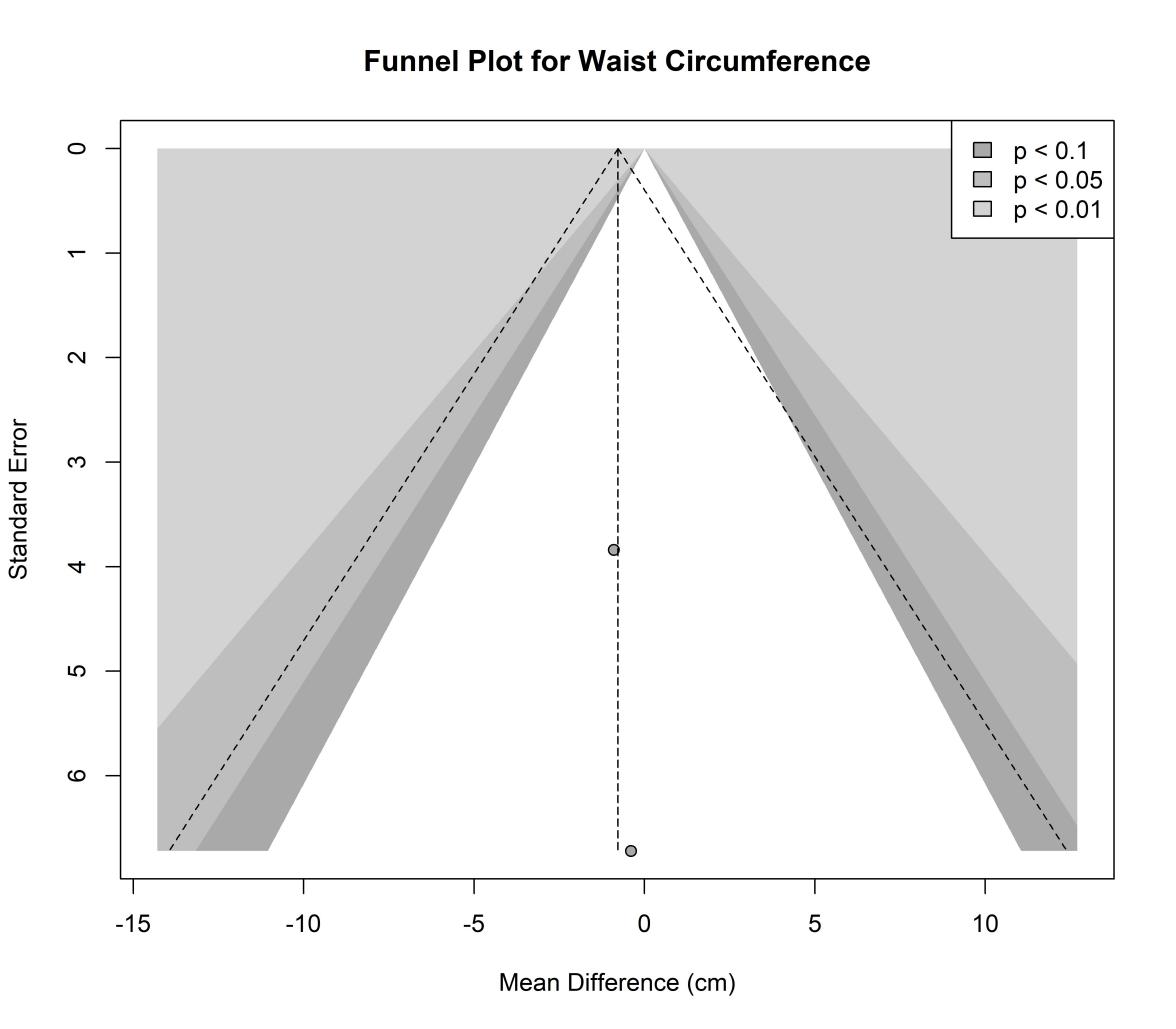


# Figures S19-S31. Funnel plots for the assessment of publication bias. Each plot displays the effect estimate of individual studies (x-axis) against their standard error (y-axis). A symmetrical distribution of studies around the summary effect estimate (vertical line) suggests a low risk of publication bias.
